# Comprehensive Genomic Investigation of Tigecycline Resistance Gene *tet*(X4)-Bearing Strains Expanding among Different Settings

**DOI:** 10.1128/spectrum.01633-21

**Published:** 2021-12-22

**Authors:** Ruichao Li, Yan Li, Kai Peng, Yi Yin, Yuan Liu, Tao He, Li Bai, Zhiqiang Wang

**Affiliations:** a Jiangsu Co-Innovation Center for Prevention and Control of Important Animal Infectious Diseases and Zoonoses, College of Veterinary Medicine, Yangzhou University, Yangzhou, People’s Republic of China; b Institute of Comparative Medicine, Yangzhou University, Yangzhou, People’s Republic of China; c Jiangsu Key Laboratory for Food Quality and Safety-State Key Laboratory Cultivation Base of Ministry of Science and Technology, Institute of Food Safety and Nutrition, Jiangsu Academy of Agricultural Sciences, Nanjing, China; d Key Laboratory of Food Safety Risk Assessment, National Health Commission of the People’s Republic of China, China National Center for Food Safety Risk Assessment, Beijing, People’s Republic of China; The Pennsylvania State University

**Keywords:** *tet*(X4), bacteria, plasmids, food safety, genomics, tigecycline resistance

## Abstract

The emergence of plasmid-mediated tigecycline resistance genes has attracted a great deal of attention globally. Currently, no comprehensive in-depth genomic epidemiology study of *tet*(X4)-bearing pathogens present of pork origin as the One Health approach has been performed. Herein, 139 fresh pork samples were collected from multiple regions in China and 58 *tet*(X4)-positive strains were identified. The *tet*(X4) gene mainly distributed in Escherichia coli (*n* = 55). Besides, 4 novel *tet*(X4)-positive bacterial species Klebsiella pneumoniae (*n* = 2), Klebsiella quasipneumoniae (*n* = 1), Citrobacter braakii (*n* = 1) and Citrobacter freundii (*n* = 1) were first characterized here. Four different core *tet*(X4)-bearing genetic environments and five types of *tet*(X4)-bearing tandem duplications were discovered among 58 strains. The results of the phylogenetic tree showed that there was some correlation between E. coli strains from pork, human, pig farms, and slaughterhouses. A total of seven types of plasmid replicons were found in *tet*(X4)-positive plasmids, among which multireplicon plasmids were observed. Notably, two *tet*(X4)-positive fusion plasmids pCSZ11R (IncX1-IncFIA-IncFIB-IncFIC) and pCSX5G-tetX4 (IncX1-IncFII-IncFIA) were formed by IS*26* in the hot spot. Besides, six samples were identified to harbor two different *tet*(X4)-bearing strains. More interestingly, the absolute quantitative results showed that the expression levels of *tet*(X4) between different strains with different *tet*(X4) copies were approximate. In this study, the genetic environment of *tet*(X4)-positive plasmids containing different plasmid replicons was analyzed to provide a basis for the further development of effective control measures. It is also highlighted that animal-borne *tet*(X4)-bearing pathogens incur a transmission risk to consumed food. Therefore, there is an urgent need for large-scale monitoring as well as the development of effective control measures.

**IMPORTANCE** Tigecycline was considered the last-line drug against serious infections caused by multidrug-resistant Gram-negative bacteria. However, the plasmid-mediated tigecycline resistance gene *tet*(X) has been widely reported in different sources of Enterobacterales and Acinetobacter in China. China is one of the largest pig-producing nations in the world, and in-depth investigation of gene in pork is vital to figure out the fundamental dissemination of these genes and set up a reasonable control framework. In this study, we conducted an in-depth and systematic analysis of the diversity of *tet*(X4)-positive plasmids and the genetic environment of *tet*(X4) contained in pork samples from multiple regions of China, providing a basis for further development of effective control measures. It is also highlighted that animal-borne *tet*(X4)-bearing pathogens incur a transmission risk to consumed food. Therefore, there is an urgent need for large-scale monitoring as well as the development of effective control measures.

## INTRODUCTION

Recently, multidrug-resistant (MDR) and extensively drug-resistant (XDR) Gram-negative pathogens pose serious threats to public health and food security (1, 2). Tigecycline was commonly used in clinical settings since it has a broad-spectrum activity (3, 4). In 2010, tigecycline was first applied in clinical treatment for treating XDR Enterobacteriaceae in China, the overexpression of efflux pumps and mutations within the tigecycline drug-binding sites were the main resistance mechanisms (5–7). However, He et al. discovered the plasmid-mediated mobile tigecycline resistance genes *tet*(X3) and *tet*(X4) in Enterobacteriaceae and Acinetobacter in 2019, which posed a severe threat to global public health (8). Of concern, previous studies have shown that *tet*(X4) has been found to coexist with *mcr-1* or *bla*_NDM-1_ in the same strain (9, 10). The strains resistant to multiple last-resort antibiotics regarded as new superbugs may disseminate globally.

So far, *tet*(X4) has been discovered in several bacteria species such as Escherichia coli, Aeromonas caviae, Acinetobacter sp., and Escherichia fergusonii (8, 10–12). Meanwhile, the *tet*(X4) gene is widely distributed on plasmids of diverse replicon types (13). All these results illustrated that the *tet*(X4) gene has the potential to extensively disseminate and should arouse our attention. Currently, there is no systematically investigation on the transferability and fitness of *tet*(X4)-carrying strains isolated from pork samples. Here, we analyzed the emerging *tet*(X4)-bearing strains isolated from pork samples across 10 regions of China in 2019. We found multiple distinct strains carrying the *tet*(X4) gene and illustrated the complex *tet*(X4) genetic environments, showed a possibility of *tet*(X4) spreading into the different plasmids.

## RESULTS

### Prevalence of *tet*(X4) positive isolates among pork in multiple regions.

**FIG 1:**
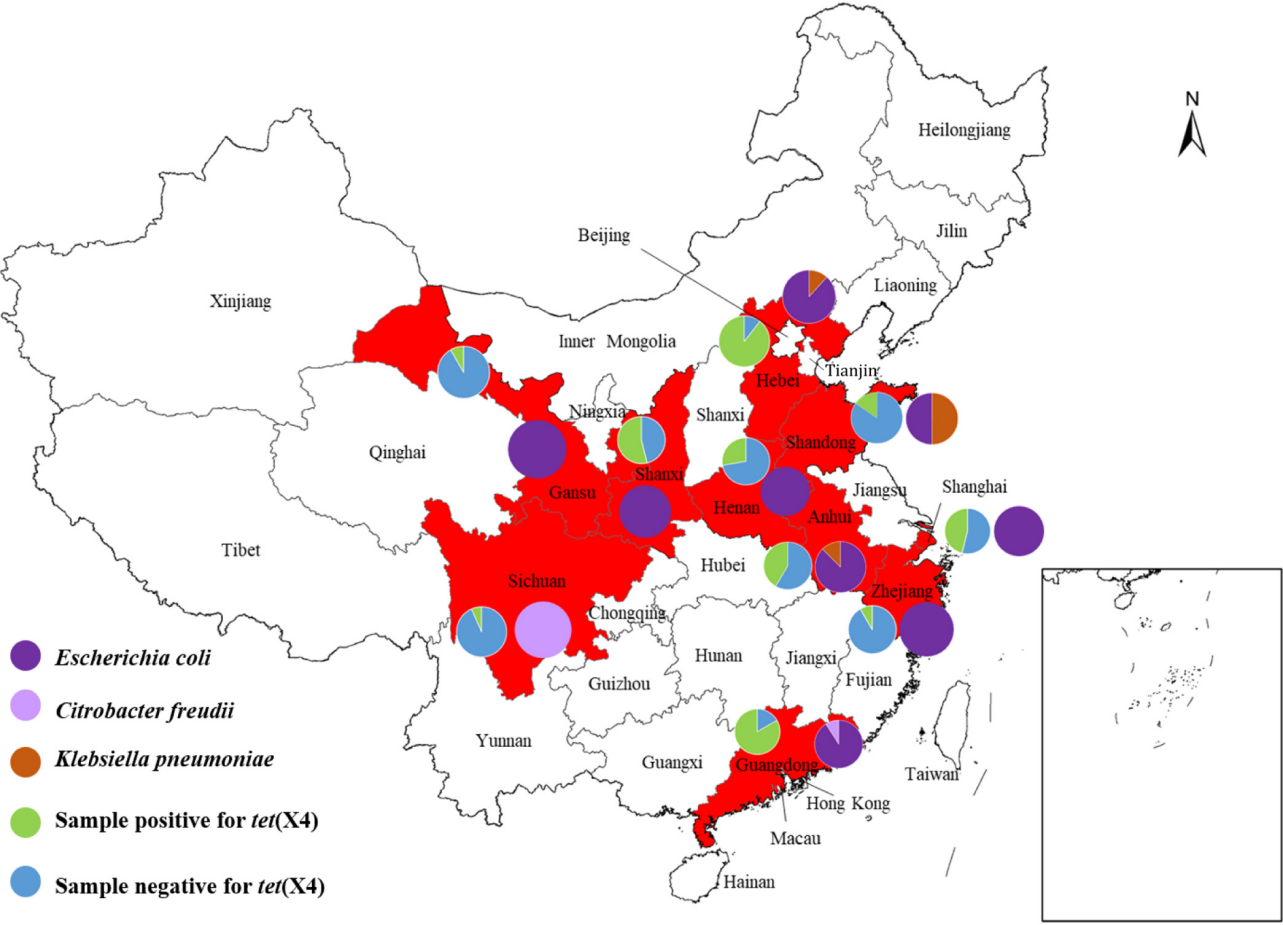
Map of the distribution of the collected retail pork in China. A total of 139 samples, 58 tigecycline-resistance strains. The red region represents the province in which *tet*(X4)-positive strains were isolated.

A total of 58 tigecycline-resistance strains were obtained from 139 samples of fresh pork. There was a difference in the positive rate of *tet*(X)-carrying bacteria among pork samples from different regions (Fig. 1). Guangdong (10/12, 83.33%), Hebei (14/19, 74.68%) and Shanxi (7/13, 53.85%) had a relatively high *tet*(X)-positive rate (Table S1). The 58 *tet*(X4)-positive strains were overwhelmingly dominated by E. coli (91.38%), followed by K. pneumoniae (3.4%), *K. quasipneumoniae* (1.7%), Citrobacter braakii (1.7%), and Citrobacter freundii (1.7%) (Table S2). To the best of our knowledge, this is the first time that *tet*(X4) has been discovered in *K. quasipneumoniae*, C. freundii *and C. braakii* in pork. Notably, a low number of samples and sampling bias may affect the positive rates observed.

### Antimicrobial susceptibility testing, resistance genes and virulence gene.

According to the result of MICs (Table S3), 58 *tet*(X4)-positive strains showed resistance to multiple drugs and were all resistant to tigecycline (8 mg/liter-64 mg/liter) and other tetracyclines (doxycycline, oxytetracycline, tetracycline, and minocycline). In addition, most of them also showed resistance to florfenicol, ceftiofur and amoxicillin. But all these strains were susceptible to meropenem and colistin. The phenotype could in most cases be explained by the carriage of the corresponding resistance genes. The *tet*(X4)-positive strains contained multiple antibiotic resistance genes (7–25), including sulfonamides (*sul* gene 45/58), aminoglycosides (*aadA*, 58/58), β-lactam (*bla*_TEM-1_ 37/58), phenicols (*floR*, 54/58), tetracyclines (*tet*[A], 48/58), trimethoprims (*drfA12* 32/58), and quinolones (*qnrS1*, 40/58). In accordance with the findings shown in Fig. S1, most of the E. coli carried a little number of virulence genes. The K. pneumoniae SDP9R strain belonged to ST1418 and carried the yersiniabactin biosynthetic gene cluster (*ybt 10 [YbST78] in the integrative conjugative element ICEKp4*). Further, we found aerobactin (*iuc* 3, AbST23) in K. pneumoniae AB4-4.

### A wide variety of *tet*(X4)-harboring plasmids.

**FIG 2:**
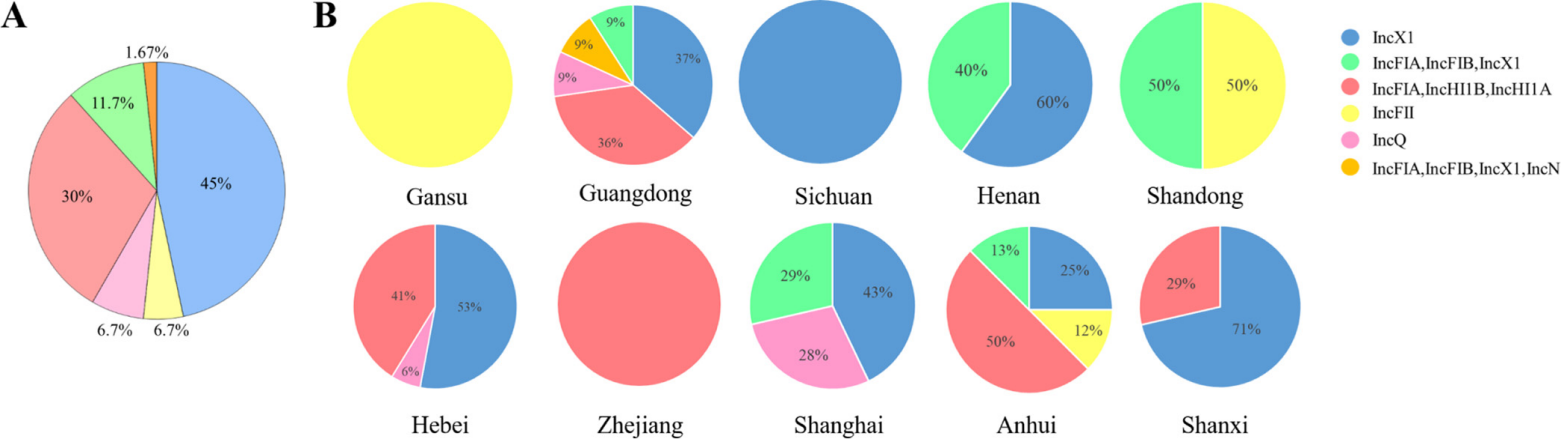
The distribution of different Inc group plasmids in all *tet*(X4)-positive strains. (A) The percentage of Inc groups found in all *tet(*X4)-positive strains. (B) The distribution of the different Inc groups in 10 regions.

In total, the 58 *tet*(X4)-harboring plasmids were classified into six plasmid replicon types. As shown in Fig. 2 and Table S4, the IncX1 and IncFIA-IncHI1B-IncHI1A type plasmids were observed to be the most prevalent. The IncX1 type plasmids were detected in multiple species and provinces, which further illustrated the widespread dissemination of IncX1 type plasmid carrying *tet*(X4). Besides, plasmids of 18 representative *tet*(X4)-positive strains were selected for Nanopore long-read sequencing. Sixteen circular *tet*(X4)-encoding plasmids were obtained from these isolates (Table S5); another two plasmids pHN13R-tetX4 and pAB12-1-tetX4 contained multiple copy numbers of the *tet*(X4)-bearing regions. As shown in Table S5, a total of six plasmid Inc types were obtained from 18 plasmids.

Among the 18 plasmids, there were eight IncX1-type plasmids with a size range of positions 31–57 kb (Fig. S2). In the NCBI database, plasmid pYY76-1-2 (CP040929) was the first discovered IncX1 type plasmid carrying the *tet*(X4) gene, which was collected from cattle feces and shared high similar backbones to IncX1 plasmids in this study. The main difference between these IncX1 type plasmids was the presence or absence of type IV secretion system (T4SS) gene cluster. The IncQ type plasmid pHS2-1-tetX4 and pSH12R-tetX4 showed high plasmid-backbone similarities (BLASTN) with that of *tet*(X4)-positive plasmid pLHM10-1 (CP037909) from manure. A total of three IncFII type plasmids were discovered from these 18 plasmids and one of them is a subtype (IncFII[pCRY]) of IncFII plasmid type. IncFII(pCRY) type plasmid pSDP9R-tetX4 which was collected from K. pneumoniae SDP9R showed high similarity with *tet*(X4)-negative plasmid pKP18-3-8-IncFII (MT035876) from human urine by BLASTn. Besides, three kinds of hybrid plasmids (IncFIA-IncHI1B-IncX1-IncN; IncFIA-IncHI1B-IncHIA; IncFIA-IncHI1B-IncX1) were also discovered. The hybrid plasmids showed high plasmid backbones similarities (BLASTN) with *tet*(X4)-positive plasmids pG3X16-2-3 (CP038140), pYSP8-1 (CP037911), pRF10-1_119k_tetX (MT219823), respectively.

### Phylogenetic analysis.

**FIG 3:**
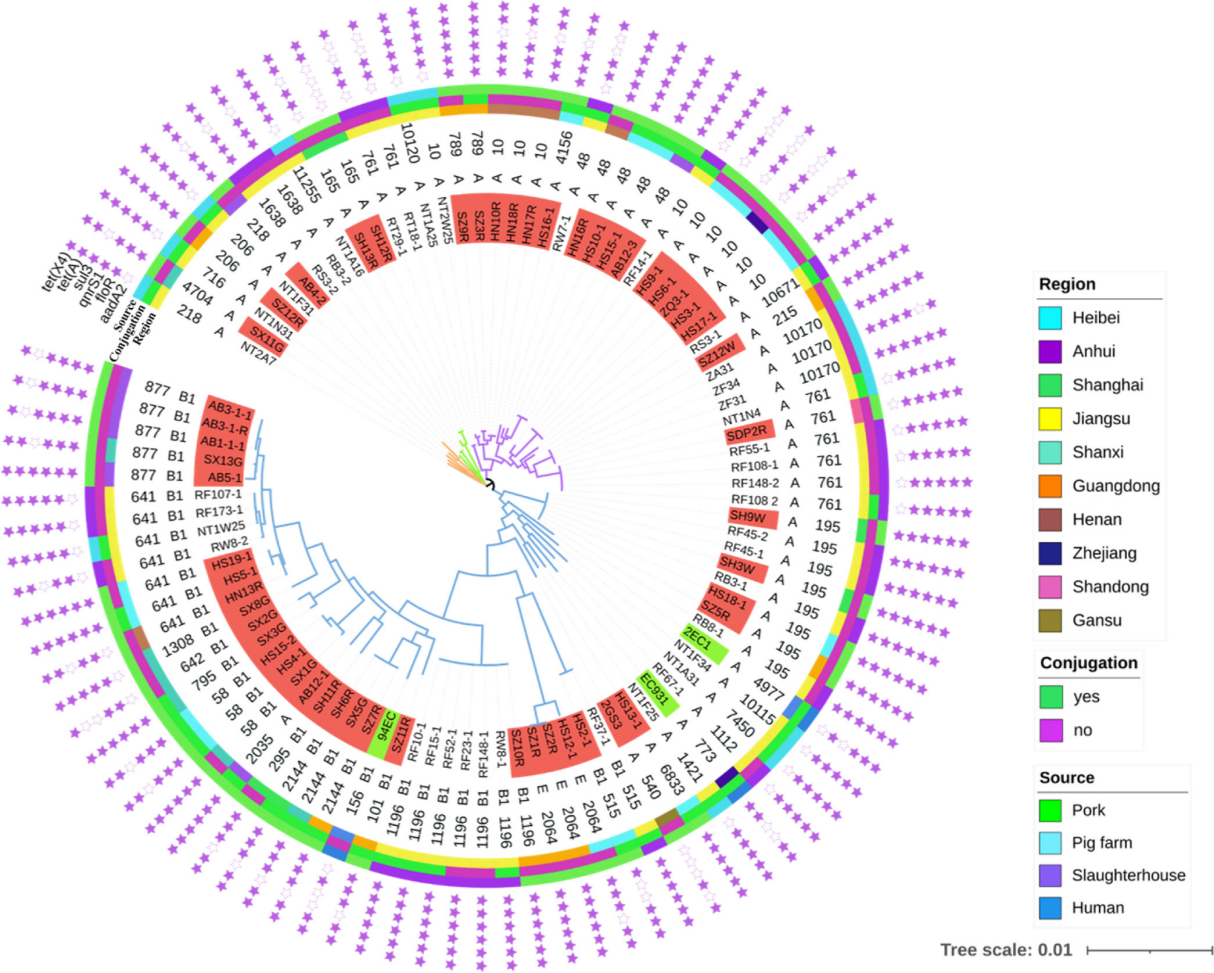
Phylogenetic tree of 96 *tet*(X4)-positive E. coli isolates from pork, human, pig farm and slaughterhouse. A total of four clusters (orange, green, purple and blue) were identified. The strains highlighted in red are from this study, green are from a human source. Resistance genes are indicated by asterisks, solid graphics indicate yes, hollow no.

To further investigate the evolutionary relationship of 53 E. coli isolated from pork samples in this study and other *tet*(X4)-positive E. coli collected from human, pig farms and porcine slaughterhouses, a phylogenetic tree was constructed. The phylogenetic tree displayed that the E. coli strains were mainly grouped into four clusters (Fig. 3, S3). Among the 53 E. coli, the most abundant phylogenetic Clermont groups were groups A (29/53, 54.72%) and B1 (21/53 39.62%), whereas groups E (5.66%) was rare (Table S6). Besides, due to the blast comparison analysis found that pCSDP9R had high similarity with the *tet*(X4)-negative plasmids in K. pneumoniae isolated from clinical and environmental conditions (Fig. S4). So, a phylogenetic tree was constructed to analyze the genetic relationship between K. pneumoniae isolated from clinical or environmental, and the results showed that they are far related (Fig. S5).

**FIG 4:**
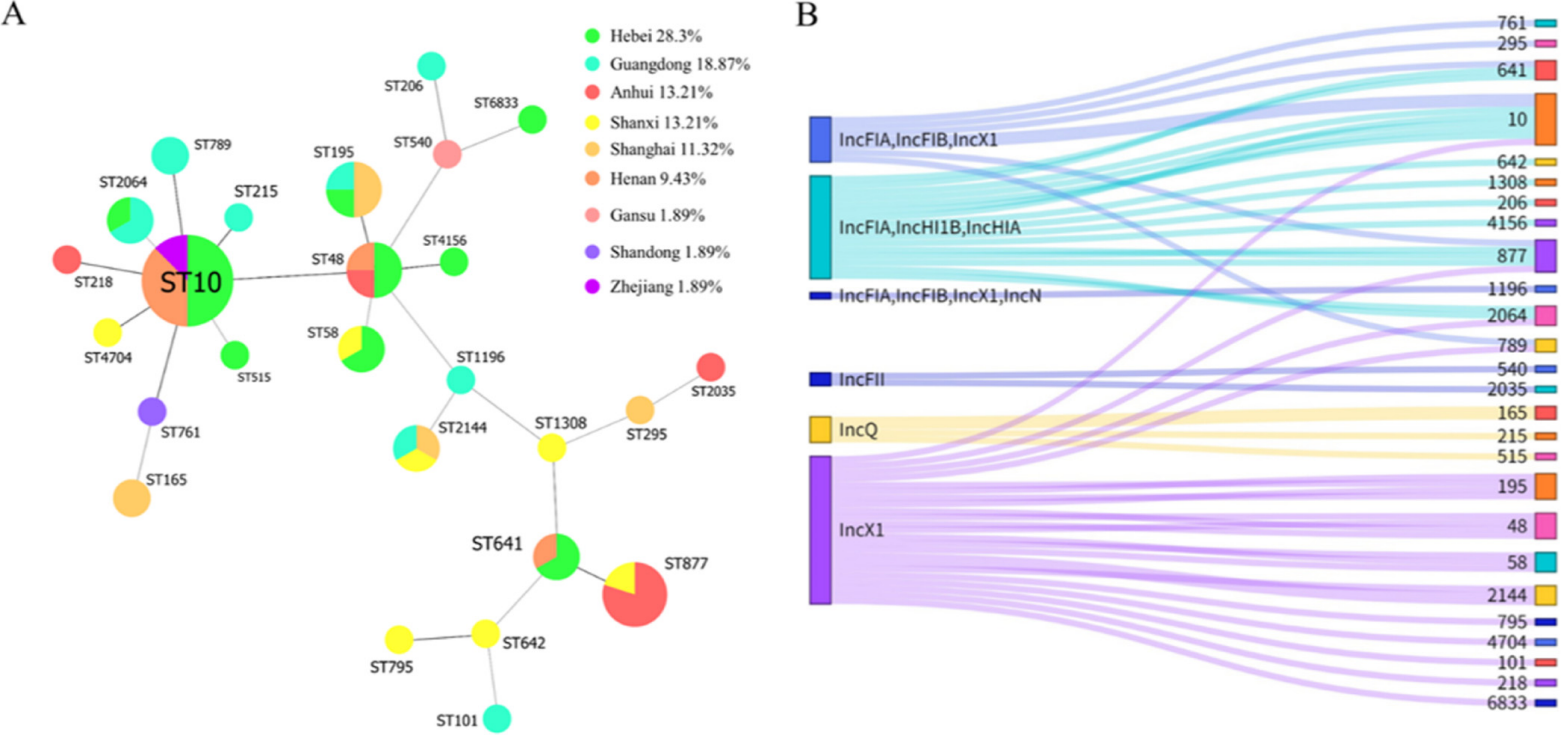
Phylogenetic trees of E. coli MLST and Sankey diagram. (A) Core genome MLST allelic profiles of E. coli. Guangdong and Hebei provinces with the most kinds of ST type. (B) Sankey diagram demonstrating the *tet*(X4)-positive E. coli ST types and the plasmid Inc type. The diameter of the line is proportional to the number of isolates, which is also labeled at the consolidation points.

According to the results of *tet*(X4)-positive strains MLST types, there were 26 distinct sequence types (STs) for E. coli, two ST types for K. pneumoniae, one ST type for *C. braakii*, one ST type for C. freundii, and one ST type for *K. quasipneumoniae*. The STs of E. coli were more diverse, with four main types (Fig. 4A), ST10 (8/53, 15.09%), ST48 (4/53, 7.5%), ST195 (4/53, 7.5%) and ST877 (5/53, 9.43%). Besides, same Inc type *tet*(X4)-positive plasmids could be identified not only from E. coli of the same ST types but also from the different ST types, which indicated the *tet*(X4) gene may spear between different ST type E. coli by plasmid horizontal transfer (Fig. 4B).

### Transmissibility of *tet*(X4)-positive plasmids.

Plasmids that carried *tet*(X4) from 22 strains were successfully transferred to E. coli C600. Besides, the conjugation frequency range of transconjugants was ranged from 10^−6^ to 10^−3^, with most conjugal frequencies being 10^−5^ (Table S7). The conjugation frequency of IncX1 type plasmids was among 10^−5^-10^−3^, which contains the highest conjugation frequency compared to other plasmid replication types in this study and was more likely to transfer.

The plasmids of four transconjugants were larger than the plasmids of their donor strains, except for one transconjugant plasmid pCAB12-1-tetX4 which was smaller. Compared with the donor plasmid pAB12-1-tetX4, pCAB12-1-tetX4 becomes smaller since some of the *tet*(X4) copy regions of pAB12-1-tetX4 were lost during the transfer. Opposite to this, the reason that pCHS10-1-tetX4 and pCSC4R-tetX4 become bigger were mainly the increasing of copies of the *tet*(X4) region in the transconjugants.

**FIG 5:**
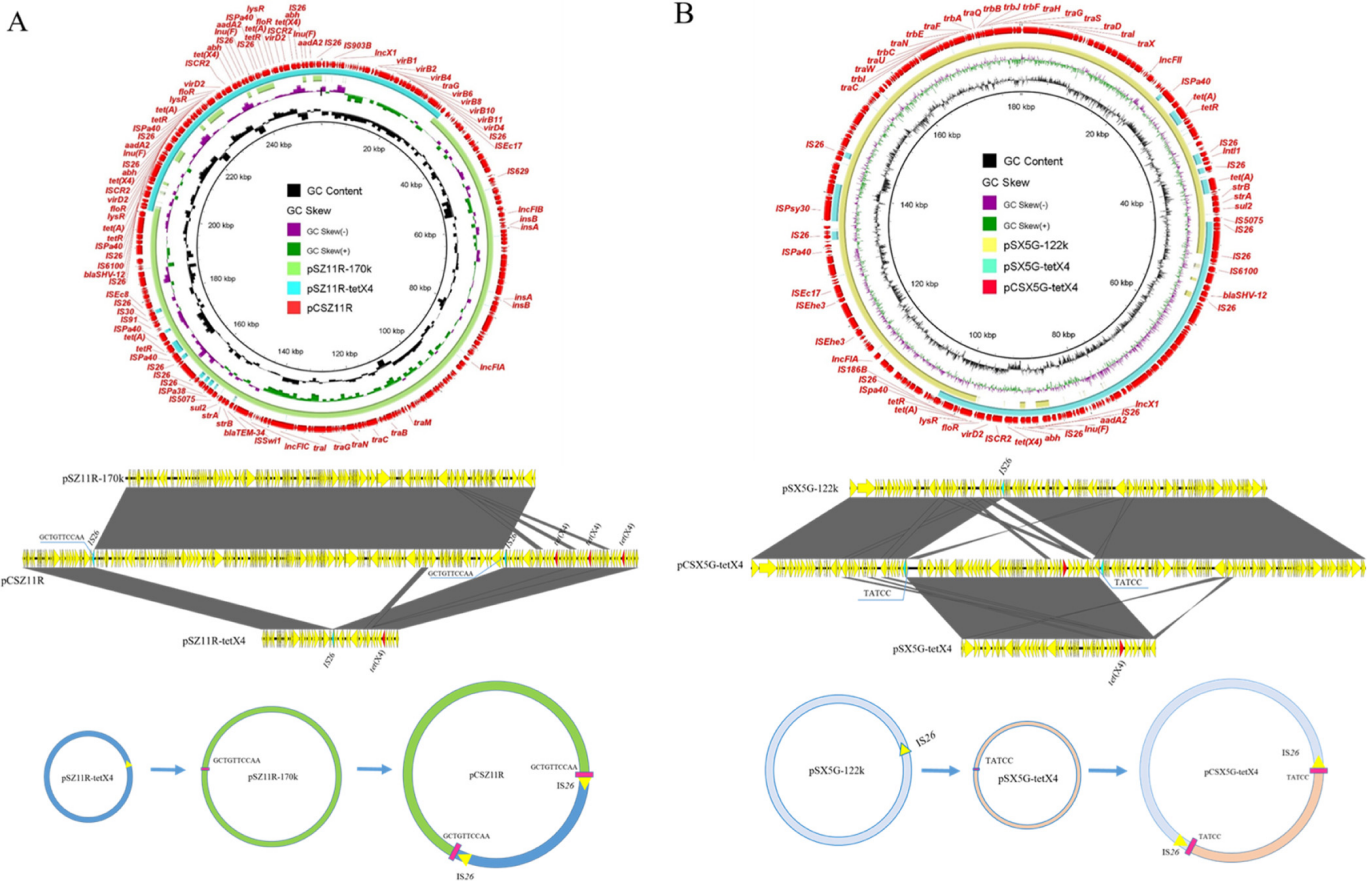
Mechanisms of fusion plasmids generation. (A) Formation mechanism of the fusion plasmid pCSZ11R. (B) Formation mechanism of the fusion plasmid pCSX5G-tetX4. The upper part denotes circular plasmid comparison between *tet*(X4)-positive plasmids in the transconjugants and their progenitor plasmids in the donor strains. The middle part denotes linear plasmid comparison between *tet*(X4)-positive plasmids and their progenitor plasmids, and the gray regions indicate the homologous region. At the bottom, proposed generation processes of two cointegrate plasmids were mediated by IS*26*.

Notably, two transconjugants CSZ11R and CSX5G showed much bigger than plasmids of the parental strain (Table S8), which mean the plasmid homologous recombination may occur (14–16). Detailed sequence analysis showed that 170 kb plasmid pSZ11R-170k and 56 kb plasmid pSZ11R-tetX4 of parental strain formed a 256 kb size fusion plasmid pCSZ11R during the conjugation. Further intensive analyses revealed that two more 13 937 bp repeat segments hp-*tetR*-*tet*(A)-*lysR*-*floR*-*virD2*-IS*CR2*-*tet*(X4)-*abh*-IS*26*-*lnu*(F)-*aadA2*-hp-IS*26*-IS*Pa40* were found in pCSZ11R compared with plasmids in SZ11R. The plasmid fusion mechanism are as follows, the insertion sequence IS*26* located in plasmid pSZ11R-tetX4 attacked the 10 bp size hot spot (GCTGTTCCAA) of pSZ11R-170k through intermolecular replicative transposition and resulted in IS*26* repetition (Fig. 5A). This plasmid fusion mechanism resulted in 10 bp site sequence duplication and IS*26* duplication. The 180 kb fusion plasmid pCSX5G-tetX4 was formed by 122 kb size plasmid pSX5G-122k and 57 kb size plasmid pSX5G-tetX4, which has a similar fusion mechanism with pCSZ11R. The insertion sequence IS*26* located in plasmid pSX5G-122k attacked the 5 bp size hot spot (TATCC) of pSX5G-tetX4 through intermolecular replicative transposition and resulted in IS*26* repetition (Fig. 5B).

### The diversity of *tet*(X4)-harboring contexts and tandem repeats.

**FIG 6:**
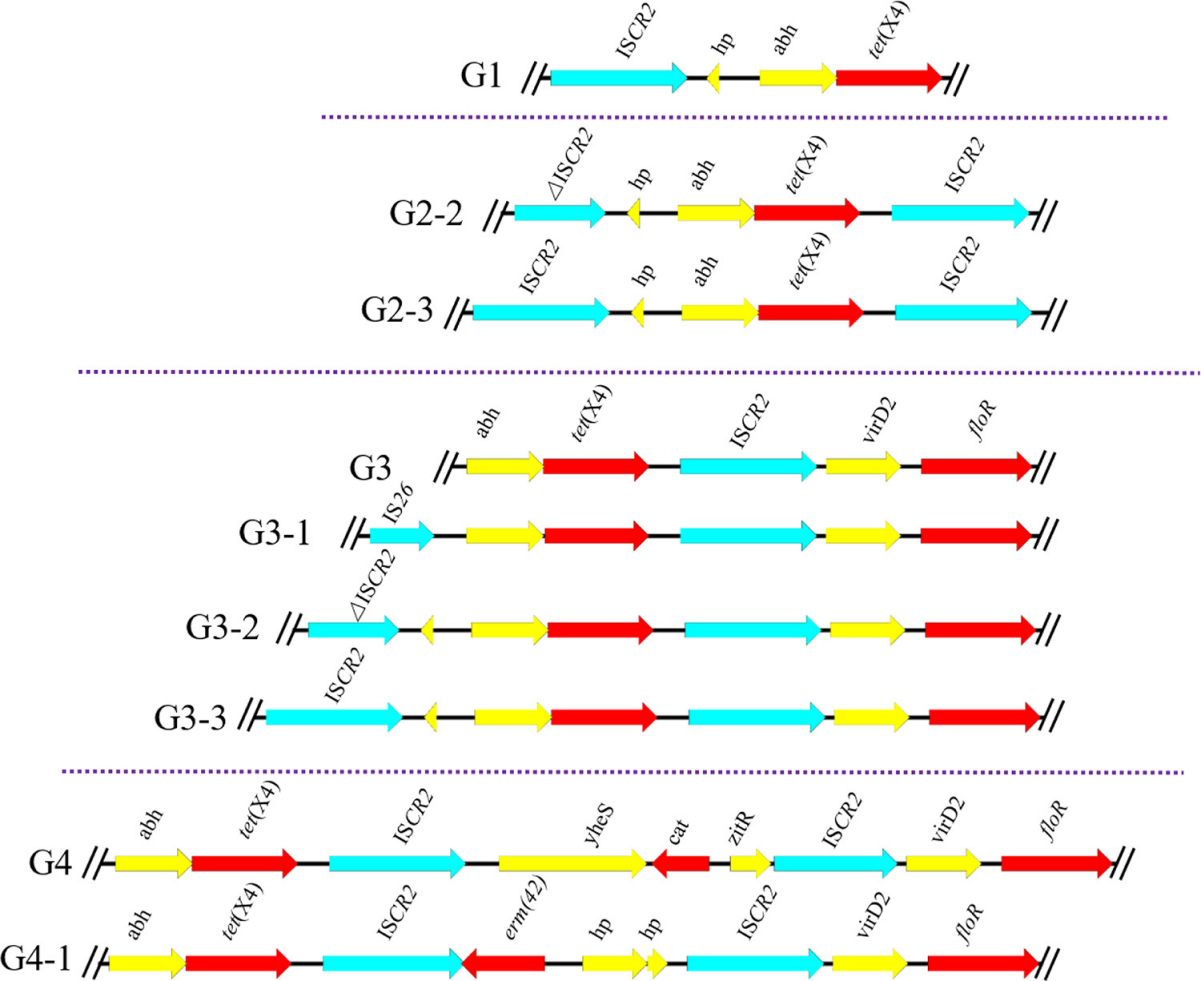
Different types of genetic environments of *tet*(X4) genes. Major types of *tet*(X4)-bearing genetic contexts among the 58 *tet*(X4)-bearing plasmids.

**FIG 7:**
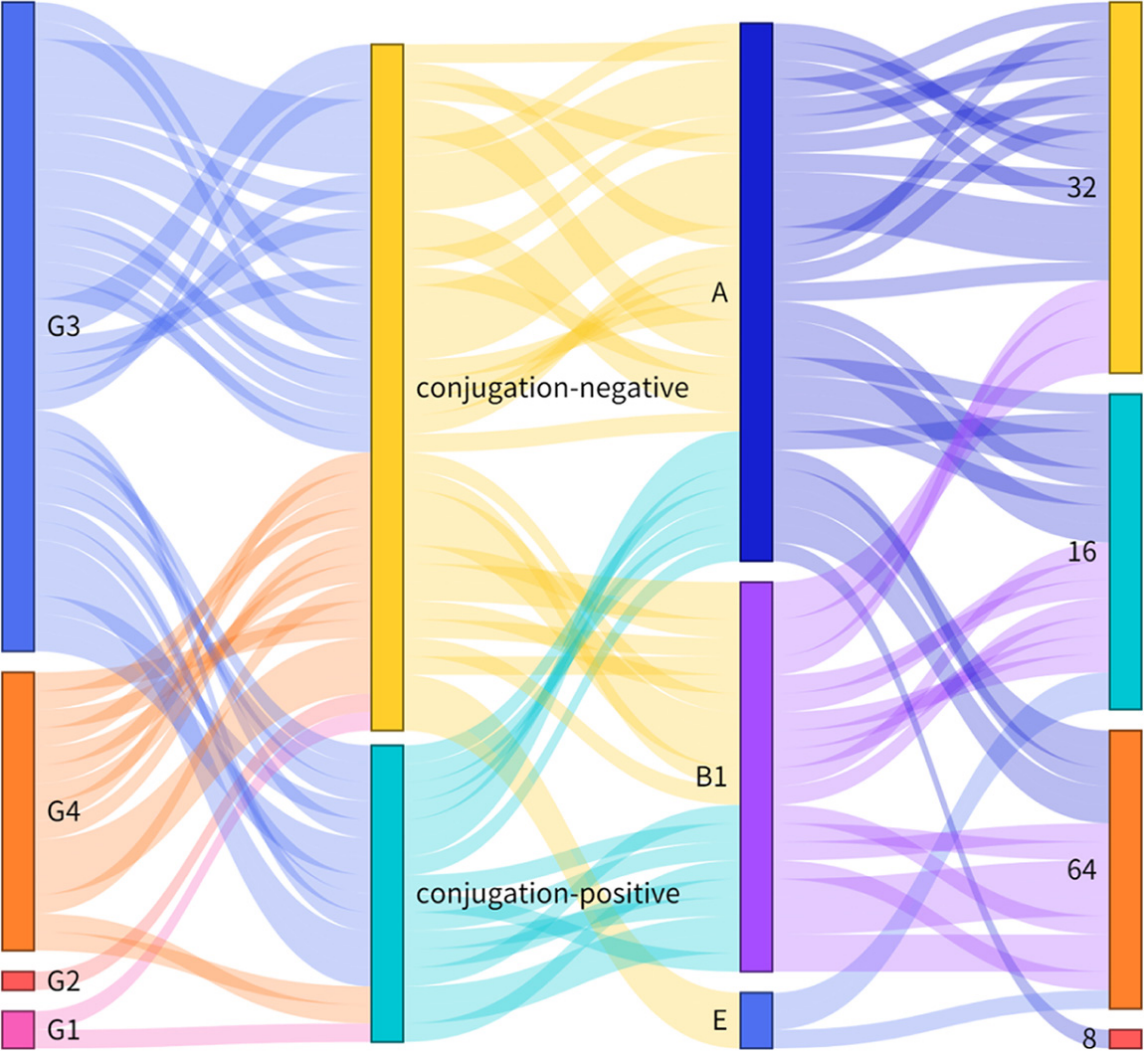
Sankey diagram combining the genetic environments bearing *tet*(X4), conjugation, E. coli group and the MIC for tigecycline. The diameter of the line is proportional to the number of isolates, which is also labeled at the consolidation points.

The genetic environments of *tet*(X4) can be categorized into four groups (Fig. 6). The G1 (*n* = 2) can be classified as group 1, compared with the structure IS*CR2*-ORF2-*abh*-*tet*(X4)-IS*CR2* of original *tet*(X4)-carried plasmid p47EC (MK134376), G1 absent the *tet*(X4) downstream region IS*CR2*. Analyzed of group 2 (G2-2, *n* = 1; G2-3, *n* = 1), the *tet*(X4) upstream region of G2-2 is ΔIS*CR2* gene, which is the main difference between the *tet*(X4) genetic environment of p47EC. Group 3 (G3, *n* = 22; G3-1, *n* = 12; G3-2, *n* = 2 G3-3, *n* = 1) had the conserved structure *abh*-*tet*(X4)-IS*CR2*-*virD2*-*floR*, the difference between them is the upstream region with different gene (IS*CR2*, ΔIS*CR2*, IS*26*). The last group G4 (G4, *n* = 16; G4-1, *n* = 1) had two longest genetic region, *abh*-*tet*(X4)-IS*CR2*-*yheS*-*cat*-*zitR*-IS*CR2*-*virD2*-*floR* and *abh*-*tet*(X4)-IS*CR2*-*erm*(26)-orf-orf-IS*CR2*-*virD2*-*floR*. All four *tet*(X4) genetic environments were further analyzed by combined with the transmissibility, E. coli Phylogenetic group and the MIC of tigecycline (Fig. 7). The result displayed various and complex genetic environments of *tet*(X4)-positive E. coli during the *tet*(X4) gene spreading.

A total of five types repeat regions were discovered (Fig. S6), two repeat regions were detected in original plasmids (pHN13R-tetX4, 4 copy; pAB12-1-tetX4, multiple copy), and the others 3 kinds (pCSZ11R, 3 copy; pCHS10-1, 2 copy; pCSC4R, 4 copy) were found in transconjugants (pCSZ11R: IS*PA40*-*tetR*-*tet*(A)-*lysR*-*floR*-*virD2*-IS*CR2*-*tet*(X4)-*abh*-IS*26*-*lnu*(F)-*aadA2*-IS*26* 13,879 bp; pCHS10-1: *tet*(X4)-*abh*-IS*26*-*lnu*(F)-*aadA2*-IntI1-IS*26* 7,445 bp; pCSC4R: *tet*(X4)-*abh*-IS*CR2*-orf-IS*CR2* 6,926 bp).

### Fitness cost and plasmid stability.

It is interesting to note that all three transconjugants contained a single plasmid showed equivalent growth rates to C600 (*P value* > 0.5) (Fig. S7A). Meanwhile, to compare the biofilm-forming abilities of *tet*(X4)-harboring transconjugants of different plasmid replicon types, we performed a biofilm assay. As shown in Fig. S7B, the transconjugant with pCSDP9R, pCAB12-1 or pCSC4R showed no difference in biofilm formation from the plasmid-free recipient strain C600.

In order to evaluate the stability of *tet*(X4) gene and *tet*(X4)-positive plasmids of different plasmid replicon types in the strains and their transconjugants, samples from the 10th, 20th, and 30th generations were selected for analysis. Under the pressure of tigecycline, the *tet*(X4) gene and *tet*(X4)-positive plasmid were inherited stably during the passage of strains (SC4R, SDP9R, AB12-1) and transconjugants (CSC4R, CSDP9R, CAB12-1). According to Fig. S8, *tet*(X4) was found in all 10th generation strains and transconjugants, clones negative for *tet*(X4) were detected in the 20th and 30th generation strains and transconjugants in the absence of tigecycline exposure. Most of the *tet*(X4) gene loss resulted from the loss of the plasmid carrying the *tet*(X4) gene. Loss of the *tet*(X4) gene in both strains SDP9R and CSDP9R resulted from a loss of the *tet*(X4)-positive plasmid.

To further explore the mechanism of *tet*(X4) loss but plasmid presence, plasmids of the single clone were picked out for Nanopore sequencing. Results are shown in Fig. S9, the comparison between the plasmids pAB12-1-tetX4 and pAB12-1-ΔtetX4 showed that the 77 kb region, including three 12 kb same regions IS*CR2*-*tet*(X4)-*abh*-*dgkA*-IS*CR2*-*lysR*-*floR*-*virD2*-IS*CR2* and three 8 kb same regions ΔIS*CR2*-*erm*(26)-IS*CR2-tet*(X4)-*abh*-*dgkA*-ΔIS*CR2* was lost in pAB12-1-ΔtetX4. Detailed analysis of the transconjugants pCAB12-1 and pCAB12-1-ΔtetX4 showed that pCAB12-1-ΔtetX4 lost the 24 kb region compared with pCAB12-1, which contained multiple resistance genes such as *sul2*, *floR* and *tet*(X4) gene. Besides, compared with pSC4R-tetX4, the plasmid pSC4R-ΔtetX4 lost the 27 kb region, including four 7 kb same regions IS*CR2*-*tet*(X4)-*abh*-ΔIS*CR2*. And the comparison between the transconjugants plasmids pCSC4R and pCSC4R-ΔtetX4 indicated that the 7 kb region was lost in pCSC4R-ΔtetX4.

### Copy numbers change.

To further analyze the relationship between IS*CR2* and *tet*(X4), the contact of copy number between IS*CR2* and *tet*(X4) was analyzed by Illumina sequencing data. We found that 49 (84.48%) of 58 *tet*(X4)-positive strains contained one copy of *tet*(X4), and 9 (15.52%) of 58 possessed two or more copies of *tet*(X4). And the copy number of IS*CR2* was always greater than or equal to the number of *tet*(X4) in the strain (Fig. S10). Besides, the copy number change of *tet*(X4) and IS*CR2* gene in CSZ11R and 30th passaged strains was also analyzed by Nanopore sequencing raw reads. The number of *tet*(X4) and IS*CR2* genes in 30th passaged strains were all increased compared with CSZ11R. More interestingly, the copy number of IS*CR2* on the same contig is always more than or equal to the *tet*(X4) gene.

To explore the absolute expression of *tet*(X4) in different strains that contain different numbers *tet*(X4), high linearity (R^2^ ≥0.997) standard curves were recovered by qRT-PCR assay (Fig. S11A). Expression levels of *tet*(X4) gene between different strains which contained different number *tet*(X4) gene was approximate (Table S9). Notably, the expression of *tet*(X4) gene in these strains with 4 mg/liter tigecycline was increased slightly than that without strains. Besides, the relative expression of *tet*(X4) gene (Fig. S11B) according to the result of relative quantification, rising with the concentration of tigecycline.

## DISCUSSION

In this study, we isolated *tet*(X4)-bearing strains in pork samples from various areas in China and conducted a comprehensive molecular typing study. A multitude of different *tet*(X4)-positive strains have been isolated from pork samples. Although the *tet*(X4)-positive E. coli have been reported in several articles (17–19), to the best of our knowledge, this is the first study that *tet*(X4) gene has been identified in K. pneumoniae, *K. quasipneumoniae*, *C. braakii,* and C. freundii from pork samples. Besides, the *tet*(X4)-positive plasmids in this study were mainly found in E. coli, indicated that E. coli is a huge reservoir of *tet*(X4)-positive plasmids. All the *tet*(X4)-carrying strains exhibited high resistance to tigecycline (8 mg/liter-64 mg/liter), and they also confer resistance to multiple classes of antibiotics, which could bring great difficulty to the clinic treatment.

Several reports indicate that IS*26* could mediate the fusion of other plasmids to form MDR plasmids (20, 21). This suggested that IS*26* played an important role in the transfer of *tet*(X4) and may promote the evolution of the *tet*(X4)-positive plasmids. The plasmids size changes due to the *tet*(X4) copy number variation during the transfer experiments, imply the number of *tet*(X4) may not stable during the conjugation assay. Furthermore, the tandem repeated regions of *tet*(X4) were also found in two parental strains that were different from the previous article (8, 13, 22, 23). Unlike the tandem duplication of other genes (24), the MICs of *tet*(X4) tandem repeat strains to tigecycline were not increased significantly, which is a strange phenomenon that needs us to further investigations.

The phylogenetic tree displayed E. coli strains from pork samples, human source samples, pig farms and porcine slaughterhouses are not in independent clade, which implies that *tet*(X4)-positive E. coli may spread along the entire production chain and pose a great threat to human health care and animal food production. Despite the ST type of *tet*(X4)-positive E. coli isolated from human source is different from other *tet*(X4)-positive E. coli in this study, the IncX1 type plasmid carrying the *tet*(X4) gene in strain EC931 that isolated from human has a high similarity with the same Inc type plasmid carrying the *tet*(X4) gene in this study. Besides, the *tet*(X4) gene from strain 2EC1 that collected from human source was located on a conjugative plasmid. This phenomenon indicated that *tet*(X4) in human and pig sources may have the risk of mutual transmission through plasmids. It has been proved in previous literature that the phylogroups A and B1 could exhibit an increased drug resistance pattern (25). Besides, the phylogenetic groups of *tet*(X4)-positive E. coli in pork, pig farms and pig slaughterhouses were mainly group A, followed by group B1. It has previously been shown that E. coli isolated from human feces is mainly group A, while it is mainly group B1 in animals (27, 28). In this study, E. coli belonged to group A carrying multiple drug resistance genes may be more likely to infect staff involved in the pig production chain. Moreover, the diversity of MLST types showed that the *tet*(X4)-carrying E. coli strains isolated from pork, pig farm and slaughterhouse were diverse, indicating that plasmids may play an important role in the spread of *tet*(X4) genes among E. coli.

Unlike other tandem repeat resistance genes (24, 29), the increased copy number of *tet*(X4) did not enhance the resistance of the strains to tigecycline (13, 30). The expression of *tet*(X4) carried by different *tet*(X4) copy number strains showed no significant difference according to the result of absolute quantitative, which illustrated that the sensitivity of strains to tigecycline with different *tet*(X4) copy number has no change and the contribution from *tet*(X4) amplification can be omitted. The increase in *tet*(X4) copy number with increased tigecycline concentration, which illustrated that the expression of the *tet*(X4) gene could increase under the pressure of the tigecycline drug. Insertion sequence IS*CR2* belongs to IS*CR* elements and shares 65% amino acid identity with IS*CR1* (31). Until now, IS*CR2* has been found adjacent to multiple resistant genes such as *floR*, *tetA*, *tetR*, *strA*, *strB*, *tet*(X4) and *sul2* collected in multiple plasmids from different sources (13, 32). More interestingly, the copy number of IS*CR2* has always increased together with the *tet*(X4) gene. Several published reports have indicated IS*CR2* which is surrounded by *tet*(X4) could form a circular intermediate and may transfer to other plasmids and chromosomes. This circular intermediate may recognize an IS*CR2* site and then insertion there produced a new copy, thus lead to a tandem repeat of the *tet*(X4) gene.

Despite the findings, this work has several limitations. First, we only focused on the tigecycline-resistant strains by utilizing selective recovery strategy with agar plates supplemented with tigecycline. Thus, the prevalence of isolation of tigecycline resistance in each bacterial species was not available, which is a limit of this study. Second, a low number of pork samples and sampling bias may affect the positive rates observed in different regions.

In conclusion, this study conducted an in-depth analysis of pork samples from multiregion across the country, expanding our understanding of the diversity and complexity of *tet*(X4)-positive plasmids in pork. The *tet*(X4) gene was found carried by a variety of Gram-negative bacteria and different plasmid types, which greatly increase the risk of *tet*(X4) transmission. Multiple and complex *tet*(X4) genetic environments expands their host range and poses a serious threat to human health and food safety. The insertion sequences IS*26* and IS*CR2* play an important role in plasmid fusion and drug resistance genes transfer, which suggested more attention should be paid to the role of these mobile genetic elements. Besides, effective and reasonable measures should be formulated to ensure the safety of the pork production chain.

## MATERIALS AND METHODS

### Bacterial isolates.

The fresh pork samples were randomly collected from local supermarkets and retail stores across nine provinces (Shanxi, Shandong, Sichuan, Guangdong, Gansu, Henan, Anhui, Hebei, Zhejiang) and one municipality (Shanghai) of China. Tigecycline resistant Enterobacteriaceae were selected on MacConkey agar plates containing tigecycline (4 mg/liter). Bacterial species identifications of purified strains were performed using the 16S rRNA gene sequencing. The *tet*(X4) resistance gene was determined by PCR with reported primers (8).

### Antimicrobial susceptibility testing.

The MICs of *tet*(X4)-positive strains against 13 antibiotics were conducted by broth microdilution using 96-well plates. E. coli ATCC 25922 was used as the quality control strain. Resistance breakpoint was interpreted according to the EUCAST criteria (>2 mg/liter) for tigecycline and CLSI guidelines (33) for the remaining antibiotics.

### Conjugation experiments.

Transferability of *tet*(X4) was determined by filter mating conjugation experiments using *tet*(X4)-positive strains as the donor strains and rifampicin-resistant E. coli C600 (Rif^R^) as the recipient (1:4) at 37°C. The transconjugants were recovered on LB agar plates containing tigecycline (4 mg/liter) together with rifampin (300 mg/liter). PCR and S1-PFGE were used to further confirm the transconjugants. Transfer frequencies were calculated as the number of transconjugants/total number of recipients.

### Whole-genome sequencing.

The genomes of tigecycline resistant strains were extracted with the TIANamp Genomic DNA kit (TianGen, Beijing, China) and quantified by Qubit 4 Fluorometer. Then the genomic DNA samples were sequenced using Illumina Hiseq 2500 platform. The paired-end reads were *de novo* assembled using SPAdes version 3.14.0. According to phylogenetic analysis and resistant phenotypes, 18 representative isolates were selected for further sequencing by long-read Nanopore sequencing. Complete genome sequences were obtained using Unicycler version 0.4.8 with the default parameters (34). For MDR regions that could not be resolved by short-read data or even hybrid assembly method, long reads assembly tool Flye version 2.4.2 was used to confirm the accurate structures of complex MDR regions in genomes (35).

### Bioinformatics analysis.

The assembled sequences were annotated through RAST online server (https://rast.nmpdr.org/) automatically. ResFinder, PlasmidFinder and ISfinder were used to detect the antibiotic resistance genes (ARGs), plasmid replicon types and insertion sequences (36–38). For each *tet*(X4) carrying strain that was only sequenced with the second-generation sequencing technique, the contigs acquired by Illumina sequencing of them were aligned with *tet*(X4)-positive circular plasmids carrying different replicons to obtain the *tet*(X4)-positive plasmid types (39). Virulence genes were determined using the ABRicate (https://github.com/tseemann/abricate) and Kleborate (https://github.com/katholt/Kleborate). BRIG and Easyfig were used to display plasmid comparison maps (40, 41). Multilocus sequence type (MLST) of all *tet*(X4)-positive isolates were assigned using the mlst tool (https://github.com/tseemann/mlst). The core genome MLST allelic profiles of E. coli was built using PHYLOViZ (42). The phylotyping of E. coli was performed using clermont.py software (https://github.com/A-BN/ClermonTyping). Phylogenetic trees of E. coli and K. pneumoniae were constructed using Roary and FastTree based on single nucleotide polymorphisms (SNPs) of core genomes (26, 43). The resulting phylogeny was visualized and retouched using iTol (https://itol.embl.de).

### Fitness cost of *tet*(X4)-positive plasmids.

To investigate the fitness cost of *tet*(X4)-positive plasmids, growth curves for the E. coli strain C600 and transconjugants with different *tet*(X4)-positive plasmids were performed in 96-well flat-bottom plates. E. coli strain C600 and different transconjugants bearing diverse *tet*(X4)-positive plasmids were inoculated in a test tube containing 5 ml of LB broth, and shaken cultures at a constant temperature, 37°C for 24 h. 200 μl of each culture were then added in triplicate to 96-well flat-bottom plates every 1 h to test OD600 and continue to culture the remaining bacteria. Growth curves were plotted using GraphPad Prism software.

### Biofilm formation.

The E. coli strain C600 and three transconjugants with diverse *tet*(X4)-positive plasmids were inoculated into 10 ml test tubes containing 5 ml of LB broth and then incubated overnight at 37°C. 200 μl of each culture were then added in triplicate to 96-well flat-bottom plates and incubated at 37°C for 24 h. The cells were washed twice with phosphate-buffered saline (PBS) and stained with 0.1% crystal violet for 20 min at room temperature. The wells were dried, and the bound dye was solubilized with 100 μl of 33% acetic acid for 30 min. Absorbance values were quantified by measuring the absorbance at 570 nm. A well containing sterile LB without bacteria served as the negative control. Each experiment was performed in duplicate and repeated three times.

### Plasmid stability and evolution of *tet*(X4) duplications.

Strains and corresponding transconjugants of different plasmid replicon types were grown on LB agar overnight, then single clones were randomly selected to passage for 15 days in LB broth medium with or without antibiotic pressure. 96 single clones of 10th, 20th and 30th passages were picked out separately, and the presence of *tet*(X4) and plasmid replicon was validated by PCR. To explore the copy numbers changes in passaged strains, strain CSC4R was tested under the pressure with tigecycline. Single clones were picked out from 30th generation bacteria with tigecycline. The MIC values of strains to tigecycline were tested by broth microdilution and Nanopore sequencing was used to visualize changes in *tet*(X4) copy number.

### Detection of copy numbers and expressions of *tet*(X4) by real-time PCR.

The qPCR target genes were amplified using the following forward and reverse primers: 16s-E. coli F: CCTACGGGAGGCAGCAG and R: ATTACCGCGGCTGCTGG TetX4 F: ATAATTGGTGGTGGACCCGT and R: AATTCTTGCCTCTCGGTCGT. Plasmid pCE2 containing *tet*(X4) was used to generate standard curves. Strains (SZ11R, CSZ11R, CSC4R) contained different *tet*(X4) numbers were used as samples and incubated with or without tigecycline (4 mg/liter). The copy numbers of plasmid DNA per microliter were calculated using the following formula, copies of 1 μl = 6.02 × 10^23^ × DNA mass concentration (ng/μl) ×10^−9^/(plasmid vector size + amplicon size bp) * 660. Besides, the relative expression of *tet*(X4) was also measured, with the gene 16s-E. coli serving as the internal control. Different concentrations of tigecycline were used to act on *tet*(X4) gene single-copy strain DH5α, which was constructed by chemical transformation. Each reaction was run in triplicate.

### Data availability.

The sequences obtained in this paper have been deposited in the GenBank database under BioProject number PRJNA665928. The sequences of transconjugants with only Nanopore data analyzed individually were deposited in the figshare database (https://figshare.com/s/fce84af26b4ee188503d) for reference.
