# Supplementary material for: Comprehensive Genomic Investigation of Tigecycline Resistance Gene tet(X4)-Bearing Strains Expanding among Different Settings
Source: Microbiol Spectr. 2021 Dec 22;9(3):e01633-21. doi: 10.1128/spectrum.01633-21 (PMC8694195; doi:10.1128/spectrum.01633-21)
Supplement: SUPPLEMENTAL FILE 1 — Supplemental material. Download SPECTRUM01633-21_Supp_1_seq10.pdf, PDF file, 2.1 MB [file spectrum01633-21_supp_1_seq10.pdf]

Supplementary Data

Supplementary Table 1. Numbers of *tet*(X4) positive samples from pork sample and their prevalence among different regions in China.

Sources	Number of samples	Number of *tet*(X4) positive strains	Number of positive samples	Positive rates of *tet*(X4) positive samples
Shandong	13	2	2	15.38%
Shanxi	13	7	7	53.85%
Sichuan	15	1	1	6.67%
Guangdong	12	11	10	83.33%
Gansu	12	1	1	8.33%
Henan	18	5	5	27.78%
Shanghai	13	6	6	46.15%
Anhui	12	8	5	41.67%
Hebei	19	16	14	73.68%
Zhejiang	12	1	1	8.33%

Supplementary Table 2. Species and numbers of *tet*(X4) positive strains in pork samples.

Species	Number of *tet*(X4) positive strains
Escherichia coli	53
Klebsiella pneumoniae	2
Citrobacter freundii	1
Citrobacter braakii	1
Klebsiella quasipneumoniae	1

Supplementary Table 3. Antibiotic susceptibility testing (MICs, mg/L) of 58 *tet*(X4)-positive strains.

IDs[a]	Sources	Species	MLST	*tet*(X) genetic contexts	Antimicrobials												
					MEM[a]	DOX	AMX	FFC	KAN	CL	CIP	TIG	OXY	TET	MIN	CFF	ENR
SX5G	Shanxi	*E.coli*	2144	G3-1	0.06	32	>64	>64	4	≤0.125	≤0.125	16	>128	>64	>128	64	≤0.5
CSX5G	Shanxi	*E.coli*	transconjugants	G3-1	≤0.03	32	>64	>64	2	≤0.125	1	8	>128	>64	64	64	4
SX3G	Shanxi	*E.coli*	795	G3	≤0.03	32	>64	>64	4	≤0.125	≤0.125	16	>128	>64	>128	2	≤0.5
CSX3G	Shanxi	*E.coli*	transconjugants	G3	≤0.03	32	>64	>64	4	≤0.125	≤0.125	8	>128	>64	32	64	0.5
SX11G	Shanxi	*E.coli*	4704	G3	0.25	64	>64	>64	8	≤0.125	≤0.125	16	>128	>64	>128	64	1
SX2G	Shanxi	*E.coli*	642	G4	≤0.03	32	>64	>64	8	≤0.125	≤0.125	16	>128	>64	32	2	≤0.5
SX8G	Shanxi	*E.coli*	1308	G4	≤0.03	32	>64	>64	4	≤0.125	≤0.125	16	>128	>64	32	2	≤0.5
SX13G	Shanxi	*E.coli*	877	G3	0.25	32	>64	>64	4	≤0.125	0.5	16	>128	>64	16	>64	2
CSX13G	Shanxi	*E.coli*	transconjugants	G3	≤0.03	32	>64	>64	4	≤0.125	≤0.125	16	>128	>64	>128	>64	>16
SX1G	Shanxi	*E.coli*	58	G3	0.06	32	>64	>64	32	≤0.125	≤0.125	16	>128	>64	32	64	≤0.5
SDP2R	Shandong	*E.coli*	761	G3	≤0.03	32	>64	>64	4	≤0.125	≤0.125	16	>128	>64	>128	4	≤0.5
SDP9R	Shandong	*K. pneumoniae*	1418	G2-3	0.125	64	>64	64	8	0.25	0.25	32	>128	>64	>128	4	≤0.5
CSDP9R	Shandong	*E.coli*	transconjugants	G2-3	≤0.03	32	4	16	2	≤0.125	0.25	4	>128	>64	64	1	≤0.5
SC4R	Sichuan	*C. freundii*	-	G3-2	0.06	128	>64	>64	>64	≤0.125	≤0.125	64	>128	>64	>128	2	2
CSC4R	Sichuan	*E.coli*	transconjugants	G3-2	0.06	64	>64	>64	>64	≤0.125	≤0.125	16	>128	>64	>128	0.25	4
SZ12W	Guangdong	*E.coli*	215	G3	≤0.03	64	>64	>64	8	≤0.125	≤0.125	32	128	>64	>128	>64	1
SZ12R	Guangdong	*E.coli*	206	G4	≤0.03	64	>64	>64	16	≤0.125	≤0.125	32	>128	>64	128	0.5	1
SZ9R	Guangdong	*E.coli*	789	G3	≤0.03	>128	>64	>64	>64	≤0.125	2	16	>128	>64	64	0.25	1
SZ2R	Guangdong	*E.coli*	2064	G4	0.06	64	>64	>64	>64	≤0.125	≤0.125	16	>128	>64	32	0.25	1
SZ10R	Guangdong	*E.coli*	1196	G3-3	≤0.03	>128	>64	>64	16	≤0.125	32	16	>128	>64	>128	1	>16
SZ7R	Guangdong	*E.coli*	2144	G3	≤0.03	128	4	>64	>64	≤0.125	≤0.125	64	>128	>64	128	64	16

CSZ7R	Guangdong	*E.coli*	transconjugants	G3	≤0.03	32	2	>64	4	≤0.125	≤0.125	16	>128	>64	32	0.25	0.25
SZ5R	Guangdong	*E.coli*	195	G3-1	≤0.03	64	>64	>64	>64	≤0.125	≤0.125	32	>128	>64	64	>64	0.25
SZ11R	Guangdong	*E.coli*	101	G3-1	≤0.03	64	>64	>64	>64	≤0.125	≤0.125	32	>128	>64	64	0.25	0.5
CSZ11R	Guangdong	*E.coli*	transconjugants	G3-1	0.06	64	>64	>64	>64	≤0.125	≤0.125	16	>128	>64	>128	2	1
SZ6R	Guangdong	*C. braakii*	109	G4	≤0.03	64	>64	>64	8	≤0.125	≤0.125	32	>128	>64	64	0.5	0.5
CSZ6R	Guangdong	*E.coli*	transconjugants	G4	≤0.03	32	>64	>64	4	≤0.125	≤0.125	8	128	>64	32	0.25	0.25
SZ3R	Guangdong	*E.coli*	789	G3	≤0.03	64	>64	>64	>64	≤0.125	1	16	>128	>64	32	0.5	1
CSZ3R	Guangdong	*E.coli*	transconjugants	G3	≤0.03	32	2	>64	4	≤0.125	≤0.125	16	>128	>64	32	0.25	0.25
SZ1R	Guangdong	*E.coli*	2064	G4	0.06	32	>64	>64	8	≤0.125	≤0.125	16	>128	>64	64	0.25	1
2GS3	Gansu	*E.coli*	540	G2-2	≤0.03	64	>64	128	16	≤0.125	0.5	16	>128	>64	64	0.5	1
C2GS3	Gansu	*E.coli*	transconjugants	G2-2	≤0.03	16	4	8	4	≤0.125	0.125	4	128	32	4	0.25	0.25
HN18R	Henan	*E.coli*	10	G3-1	≤0.03	>128	>64	>64	64	≤0.125	≤0.125	8	>128	>64	>128	>64	0.06
HN17R	Henan	*E.coli*	10	G3-1	0.06	64	>64	>64	>64	≤0.125	≤0.125	16	>128	>64	>128	>64	0.25
HN16W	Henan	*E.coli*	48	G3	≤0.03	64	>64	>64	>64	≤0.125	≤0.125	16	>128	>64	64	32	0.25
HN13R	Henan	*E.coli*	641	G3-2	0.06	>128	>64	>64	8	≤0.125	2	64	>128	>64	>128	1	8
HN10R	Henan	*E.coli*	10	G3-1	≤0.03	64	>64	>64	>64	≤0.125	≤0.125	32	>128	>64	64	64	0.5
SH11R	Shanghai	*E.coli*	295	G3	≤0.03	32	>64	>64	8	≤0.125	≤0.125	32	>128	>64	64	1	≤0.03
SH12R	Shanghai	*E.coli*	165	G1	0.06	64	>64	32	32	≤0.125	≤0.125	16	>128	>64	32	0.5	0.5
SH13R	Shanghai	*E.coli*	165	G3	≤0.03	>128	>64	>64	>64	≤0.125	≤0.125	32	>128	>64	64	0.25	2
SH3W	Shanghai	*E.coli*	195	G3	≤0.03	>128	>64	>64	>64	≤0.125	≤0.125	32	>128	>64	>128	>64	4
SH6R	Shanghai	*E.coli*	2144	G3	≤0.03	128	4	>64	>64	≤0.125	2	64	>128	>64	128	64	8
CSH6R	Shanghai	*E.coli*	transconjugants	G3	≤0.03	64	8	>64	4	≤0.125	≤0.125	32	>128	>64	64	0.25	0.25
SH9W	Shanghai	*E.coli*	195	G3-1	≤0.03	>128	>64	>64	>64	≤0.125	≤0.125	32	>128	>64	>128	>64	>16
AB1-1-1	Anhui	*E.coli*	877	G4	≤0.125	64	>64	>64	8	≤0.125	8	32	>128	>64	128	4	32
AB12-1	Anhui	*E.coli*	2035	G4-1	≤0.125	32	>64	>64	>64	≤0.125	0.5	64	>128	>64	>256	16	2

CAB12-1	Anhui	*E.coli*	transconjugants	G4-1	≤0.125	64	16	>64	8	≤0.125	2	16	>128	>64	32	4	8
AB12-3	Anhui	*E.coli*	48	G3-1	≤0.125	64	>64	>64	8	≤0.125	0.5	32	>128	>64	64	>64	≤0.5
CAB12-3	Anhui	*E.coli*	transconjugants	G3-1	≤0.125	64	>64	>64	2	≤0.125	≤0.125	32	>128	>64	64	>64	≤0.5
AB3-1-R	Anhui	*E.coli*	877	G4	≤0.125	64	>64	>64	2	≤0.125	16	32	>128	>64	128	4	>16
AB3-1-1	Anhui	*E.coli*	877	G4	≤0.125	64	>64	>64	8	≤0.125	8	64	>128	>64	64	2	32
AB4-2	Anhui	*E.coli*	218	G3-1	≤0.125	64	>64	>64	64	≤0.125	2	64	>128	>64	64	>64	2
AB4-4	Anhui	*K. pneumoniae*	35	G4	≤0.125	64	>64	>64	>64	0.25	8	64	>128	>64	256	8	8
AB5-1	Anhui	*E.coli*	877	G3	≤0.125	64	>64	>64	8	≤0.125	1	32	>128	>64	>256	8	1
HS10-1	Hebei	*E.coli*	48	G3-1	≤0.125	64	>64	>64	8	≤0.125	0.5	32	>128	>64	256	>64	2
CHS10-1	Hebei	*E.coli*	transconjugants	G3-1	≤0.125	64	>64	>64	4	≤0.125	≤0.125	16	>128	>64	32	>64	≤0.5
HS12-1	Hebei	*E.coli*	2064	G3	≤0.125	32	>64	>64	4	0.25	0.5	64	>128	>64	256	>64	≤0.5
CHS12-1	Hebei	*E.coli*	transconjugants	G3	≤0.125	64	>64	>64	8	≤0.125	≤0.125	32	>128	>64	32	>64	≤0.5
HS13-1	Hebei	*E.coli*	6833	G3	≤0.125	64	>64	>64	8	≤0.125	0.25	32	>128	>64	64	>64	1
CHS13-1	Hebei	*E.coli*	transconjugants	G3	≤0.125	64	>64	>64	4	≤0.125	≤0.125	32	>128	>64	32	>64	1
HS19-2	Hebei	*K. quasipneumoniae*	-	G3-1	≤0.125	64	>64	>64	4	≤0.125	0.5	64	>128	>64	128	>64	1
CHS19-2	Hebei	*E.coli*	transconjugants	G3-1	≤0.125	32	>64	>64	2	≤0.125	≤0.125	16	>128	64	64	16	≤0.5
HS15-1	Hebei	*E.coli*	48	G3	≤0.125	32	>64	>64	8	≤0.125	0.5	64	>128	>64	64	>64	2
CHS15-1	Hebei	*E.coli*	transconjugants	G3	≤0.125	32	>64	>64	4	≤0.125	≤0.125	32	>128	>64	32	>64	≤0.5
HS15-2	Hebei	*E.coli*	58	G3	≤0.125	64	>64	>64	4	≤0.125	0.5	64	>128	>64	128	>64	4
CHS15-2	Hebei	*E.coli*	transconjugants	G3	≤0.125	64	>64	>64	4	≤0.125	≤0.125	32	>128	>64	32	>64	≤0.5
HS16-1	Hebei	*E.coli*	4156	G3	≤0.125	64	>64	>64	4	≤0.125	≤0.125	32	>128	>64	128	32	≤0.5
CHS16-1	Hebei	*E.coli*	transconjugants	G3	≤0.125	32	>64	>64	4	≤0.125	≤0.125	8	>128	>64	64	>64	1
HS17-1	Hebei	*E.coli*	10	G3	≤0.125	64	>64	>64	16	≤0.125	0.5	32	>128	>64	128	8	2
HS18-1	Hebei	*E.coli*	195	G3	≤0.125	64	>64	>64	4	≤0.125	≤0.125	32	>128	>64	128	>64	8
HS19-1	Hebei	*E.coli*	641	G4	≤0.125	32	>64	>64	8	≤0.125	1	64	>128	>64	256	16	32

HS2-1	Hebei	*E.coli*	515	G1	≤0.125	64	>64	32	>64	≤0.125	1	64	>128	>64	>256	>64	2
CHS2-1	Hebei	*E.coli*	transconjugants	G1	≤0.125	64	8	8	4	≤0.125	≤0.125	8	>128	>64	128	4	≤0.5
HS3-1	Hebei	*E.coli*	10	G4	≤0.125	64	>64	>64	8	≤0.125	0.5	64	>128	>64	128	>64	2
HS4-1	Hebei	*E.coli*	58	G3-1	≤0.125	64	>64	>64	4	≤0.125	0.5	64	>128	>64	256	>64	2
CHS4-1	Hebei	*E.coli*	transconjugants	G3-1	≤0.125	64	>64	>64	4	≤0.125	≤0.125	32	>128	>64	128	>64	≤0.5
HS5-1	Hebei	*E.coli*	641	G4	≤0.125	>64	>64	>64	8	≤0.125	0.5	64	>128	>64	>256	4	2
CHS5-1	Hebei	*E.coli*	transconjugants	G4	≤0.125	32	>64	>64	1	≤0.125	0.25	64	>128	>64	128	2	16
HS6-1	Hebei	*E.coli*	10	G4	≤0.125	64	>64	>64	8	≤0.125	0.25	32	>128	>64	128	4	1
HS9-1	Hebei	*E.coli*	10	G4	≤0.125	32	>64	>64	16	≤0.125	0.25	64	>128	>64	128	8	2
ZQ3-1	Zhejiang	*E.coli*	10	G4	≤0.125	64	>64	>64	16	≤0.125	0.25	32	>128	>64	128	8	2

Abbreviations: MEM, meropenem; DOX, doxycycline; AMX, amoxicillin; FFC, florfenicol; KAN, kanamycin; CL, colistin; CIP, Ciprofloxacin; TIG, tigecycline; OXY, oxytetracycline; TET, tetracycline; MIN, minocycline; CFF, ceftiofur; ENR, enrofloxacin.

Supplementary Table 4. Characteristic of *tet*(X4)-bearing plasmids in different bacterial species from the pork samples in China.

Inc type[a]	Species	*tet*(X4)-positive plasmids			Strains		
		Number	Genetic contexts	Transferability (frequency)	Phylogenetic group	MLST (No. of isolates)	Tigecycline MIC(mg/L)
IncX1	*E.coli*	23	G3, G3-1	13/23 (5.87×10^{-3}-9.95×10^{-5})	A, B1, E	10, 48 (n=4), 58 (n=3), 101, 195 (n=4), 218, 789, 795, 877, 2064, 2144 (n=3), 4704, 6833	16-64
IncFII	*E.coli*	2	G2-2, G4-1	1/2 (2.01×10^{-5})	A	540, 2035	16-64
IncQ	*E.coli*	4	G1, G3	1/4 (2.6×10^{-6})	A, B1	165 (n=2), 215, 515	16-64
IncFIA-IncFIB-IncX1	*E.coli*	7	G3, G3-1, G3-2	1/7 (8.96×10^{-5})	A, B1	10 (n=2), 295, 641, 761, 789, 877	8-64
IncFIA-IncHI1B-IncHIA	*E.coli*	16	G3, G4	2/16 (3.05×10^{-5}-3.76×10^{-5})	A, B1, E	10 (n=5), 641 (n=2), 206, 642, 877 (n=3), 1308, 2064 (n=2), 4156	16-64
IncFIA-IncFIB-IncX1-IncN	*E.coli*	1	G3-3	-	B1	1196	16
IncFII	*K. pneumoniae*	1	G2-3	4.9×10^{-6}	-	1418	32
IncFIA-IncHI1B-IncHIA	*K. pneumoniae*	1	G4	-	-	35	64
IncX1	*C. freundii*	1	G3-2	2.63×10^{-4}	-	-	64
IncFIA-IncHI1B-IncHIA	*C. braakii*	1	G4	2×10^{-4}	-	109	32
IncX1	*K. quasipneumoniae*	1	G3-1	2.69×10^{-4}	-	-	64

Supplementary Table 5. Basic information of 18 *tet*(X4)-bearing plasmids sequenced by Nanopore sequencing.

Strain	Status	Size	Inc-type	Assembly Method	Sequencing Technology	*tet*(X4) repeats	Region	Accession Number	Resistance gene
pSC4R-tetX4	complete	55 809bp	IncX1	Unicycler	Oxford Nanopore MinION, Illumina	1	Sichuan	MW940620	*aph(3')-Ia,qnrS2,floR,tet*(X4)
p2GS3-tetX4	complete	91 582bp	IncFII	Unicycler	Oxford Nanopore MinION, Illumina	1	Gansu	MW940614	*qnrS1,tet*(X4)*,sul2,bla*LAP-2
pAB4-4-tetX4	complete	193 385bp	IncFIA,IncHI1B,IncHIA	Unicycler	Oxford Nanopore MinION, Illumina	1	Anhui	MW940615	*tet*(X4)*,floR,tet*(A)*,qnrS1,bla*$_{TEM\text{-}1B}$*,aadA1*
pHS10-1-tetX4	complete	48 817bp	IncX1	Unicycler	Oxford Nanopore MinION, Illumina	1	Hebei	MW940618	*tet(A),floR,tet*(X4)*,aadA2,Inu(F), blaSHV-12*
pHS19-2-tetX4	complete	57 105bp	IncX1	Unicycler	Oxford Nanopore MinION, Illumina	1	Hebei	MW940619	*bla*$_{SHV\text{-}12}$*,tet*(A)*,floR,tet*(X4)*,Inu(F),aadA2*
pSDP9R-tetX4	complete	78 159bp	IncFII	Unicycler	Oxford Nanopore MinION, Illumina	1	Shandong	MW940621	*tet*(X4)
pSH9W-tetX4	complete	31 587bp	IncX1	Unicycler	Oxford Nanopore MinION, Illumina	1	Shanghai	MW940622	*tet*(X4)*,floR,tet*(A)*,aadA2,Inu(F)*
pSX5G-tetX4	complete	57 105bp	IncX1	Unicycler	Oxford Nanopore MinION, Illumina	1	Shanxi	MW940624	*bla*$_{SHV\text{-}12}$*,tet*(A)*,floR,tet*(X4)*,Inu(F),aadA2*
pSZ5R-tetX4	complete	31 287bp	IncX1	Unicycler	Oxford Nanopore MinION, Illumina	1	Guangdong	MW940626	*aadA2,Inu(F),tet*(X4)*,floR,tet*(A)
pSZ6R-tetX4	complete	191 434bp	IncFIA,IncHI1B,IncHIA	Unicycler	Oxford Nanopore MinION, Illumina	1	Guangdong	MW940627	*tet*(X4)*,floR,qnrS1,bla*$_{TEM\text{-}1B}$*,aadA1,lnu(G)*
pSZ11R-tetX4	complete	56 939bp	IncX1	Unicycler	Oxford Nanopore MinION, Illumina	1	Guangdong	MW940629	*bla*$_{SHV\text{-}12}$*,tet*(A)*,floR,tet*(X4)*,Inu(F),aadA2*

pSX8G-tetX4	complete	190 382bp	IncFIA,IncHI1B,IncHIA	Unicycler	Oxford Nanopore MinION, Illumina	1	Shanxi	MW940625	*tet*(X4),*floR,qnrS1,bla*$_{TEM-1B}$,*aadA1,lnu(G)*
pSZ10R-tetX4	complete	130 185bp	IncFIA,IncHI1B,IncX1,IncN	Unicycler	Oxford Nanopore MinION, Illumina	1	Guangdong	MW940628	*aadA1,aadA2,aph(3'')-Ib,aph(6)-Id,blaTEM-1,cmlA1,dfrA12,erm(42),floR,mph(A),sul3,tet(A),tet(M),tet*(X4)
pHN13R-tetX4	incomplete	169 783bp	IncFIA,IncFIB,IncX1	Unicycler	Oxford Nanopore MinION, Illumina	4	Henan	MZ054178	*aadA1,blaTEM-1,erm(42),floR,qnrS1,sul2,tet(A),tet(M),tet(X4)*
pSH12R-tetX4	complete	12 806bp	IncQ	Unicycler	Oxford Nanopore MinION, Illumina	1	Shanghai	MW940623	*tet*(X4)
pHN10R-tetX4	complete	57 096bp	IncX1	Unicycler	Oxford Nanopore MinION, Illumina	1	Henan	MW940616	*aadA2,blaSHV-12,floR,lnu(F),tet(A),tet*(X4)
pHS2-1-tetX4	complete	12 805bp	IncQ	Unicycler	Oxford Nanopore MinION, Illumina	1	Hebei	MW940617	*tet*(X4)
pAB12-1-tetX4	incomplete	174 429bp	IncFII	Unicycler	Oxford Nanopore MinION, Illumina	6	Anhui	MZ054177	*aac(3)-Iid,blaLAP-2,erm(42),floR,qnrS1,sul2,tet*(X4)

Supplementary Table 6. Clermont phylotyping of 53 strains of *E. coli*.

Strain number	Phylogenetic groups		
	A	B1	E
53	29	21	3

Supplementary Table 7. Conjugation frequencies of *tet*(X4)-positive plasmids in strains of pork origin.

Strain	Transconjugants	*tet*(X4)-positive plasmids	Replicons of *tet*(X)-bearing plasmids	Conjugation frequencies
HS5-1	CHS5-1	pHS5-1-tetX4	IncX1	3.76×10^{-5}
HS4-1	CHS4-1	pHS4-1-tetX4	IncX1	9.19×10^{-5}
SC4R	CSC4R	pSC4R-tetX4	IncX1	2.63×10^{-4}
AB12-1	CAB12-1	pAB12-1-tetX4	IncFII	2.01×10^{-5}
HS12-1	CHS12-1	pHS12-1-tetX4	IncX1	5.87×10^{-3}
SX3G	CSX3G	pSX3G-tetX4	IncX1	1.72×10^{-5}
HS19-2	CHS19-2	pHS19-2-tetX4	IncX1	2.69×10^{-4}
HS13-1	CHS13-1	pHS13-1-tetX4	IncX1	1.2×10^{-5}
SZ11R	CSZ11R	pSZ11R-tetX4	IncX1	8.22×10^{-3}
SZ3R	CSZ3R	pSZ3R-tetX4	IncFIA,IncFIB,IncX1	8.96×10^{-5}
SH6R	CSH6R	pSH6R-tetX4	IncX1	5.2×10^{-4}
HS15-2	CHS15-2	pHS15-2-tetX4	IncX1	9.1×10^{-5}
HS15-1	CHS15-1	pHS15-1-tetX4	IncX1	3.4×10^{-5}
HS16-1	CHS16-1	pHS16-1-tetX4	IncFIA, IncHI1B, IncHIA	3.05×10^{-5}
HS10-1	CHS10-1	pHS10-1-tetX4	IncX1	9.19×10^{-5}
SX5G	CSX5G	pSX5G-tetX4	IncX1	5.55×10^{-5}
SX13G	CSX13G	pSX13G-tetX4	IncX1	1×10^{-4}
SZ6R	CSZ6R	pSZ6R-tetX4	IncFIA, IncHI1B, IncHIA	2×10^{-4}
AB12-3	CAB12-3	pAB12-3-tetX4	IncX1	9.95×10^{-5}
HS2-1	CHS2-1	pHS2-1-tetX4	IncQ	2.6×10^{-6}

SD9R	CSD9R	pSD9R-tetX4	IncFII	4.9×10^{-6}
SZ7R	CSZ7R	pSZ7R-tetX4	IncX1	8.8×10^{-4}

Supplementary Table 8. Plasmids participating in reorganization in the process of conjugation.

strains	Plasmids participate in Reorganization[a]	Replicon types	Resistance genes
SZ11R	pSZ11R-tetX4[a]	IncX1	*bla*$_{SHV-12}$, *tet*(A), *floR*, *tet*(X4), *Inu(F)*, *aadA2*
	pSZ11R-170k[b]	IncFIB, IncFIC	*tet*(A), *dfrA14*, *sul2*, *strA*, *strB*, *bla*$_{TEM-34}$
CSZ11R	pCSZ11R[e]	IncX1, IncFIB, IncFIC	*bla*$_{SHV-12}$, *tet*(A), *floR*, *tet*(X4), *Inu(F)*, *aadA2*, *tet*(A), *dfrA14*, *sul2*, *strA*, *strB*, *bla*$_{TEM-34}$
SX5G	pSX5G-tetX4[c]	IncX1	*bla*$_{SHV-12}$, *tet*(A), *floR*, *tet*(X4), *Inu(F)*, *aadA2*
	pSX5G-122k[d]	IncFIA, IncFII	*tet(A)*, *qnrS1*, *drfA14*, *aadA1*, *strB*, *strA*, *sul2*
CSX5G	pCSX5G-tetX4[f]	IncX1, IncFIA, IncFII	*bla*$_{SHV-12}$, *tet*(A), *floR*, *tet*(X4), *Inu(F)*, *aadA2*, *tet(A)*, *qnrS1*, *drfA14*, *aadA1*, *strB*, *strA*, *sul2*

a, b, c, d represents the plasmids that participate in Reorganization.

e, f represents the fusion plasmid.

Supplementary Table 9. The expression levels of *tet*(X4) in SZ11R and two transconjugants with different copy numbers of *tet*(X4) according to absolute quantitative PCR.

Strains	Copy numbers of *tet*(X4)	Copy numbers of *tet*(X4) transcript/ug	Concentrations of tigecycline (mg/L)
SZ11R	1	9.62E+03	0
SZ11R		1.24E+04	4
CSZ11R	3	5.18E+03	0
CSZ11R		1.22E+04	4
CSC4R	4	9.18E+03	0
CSC4R		9.30E+03	4

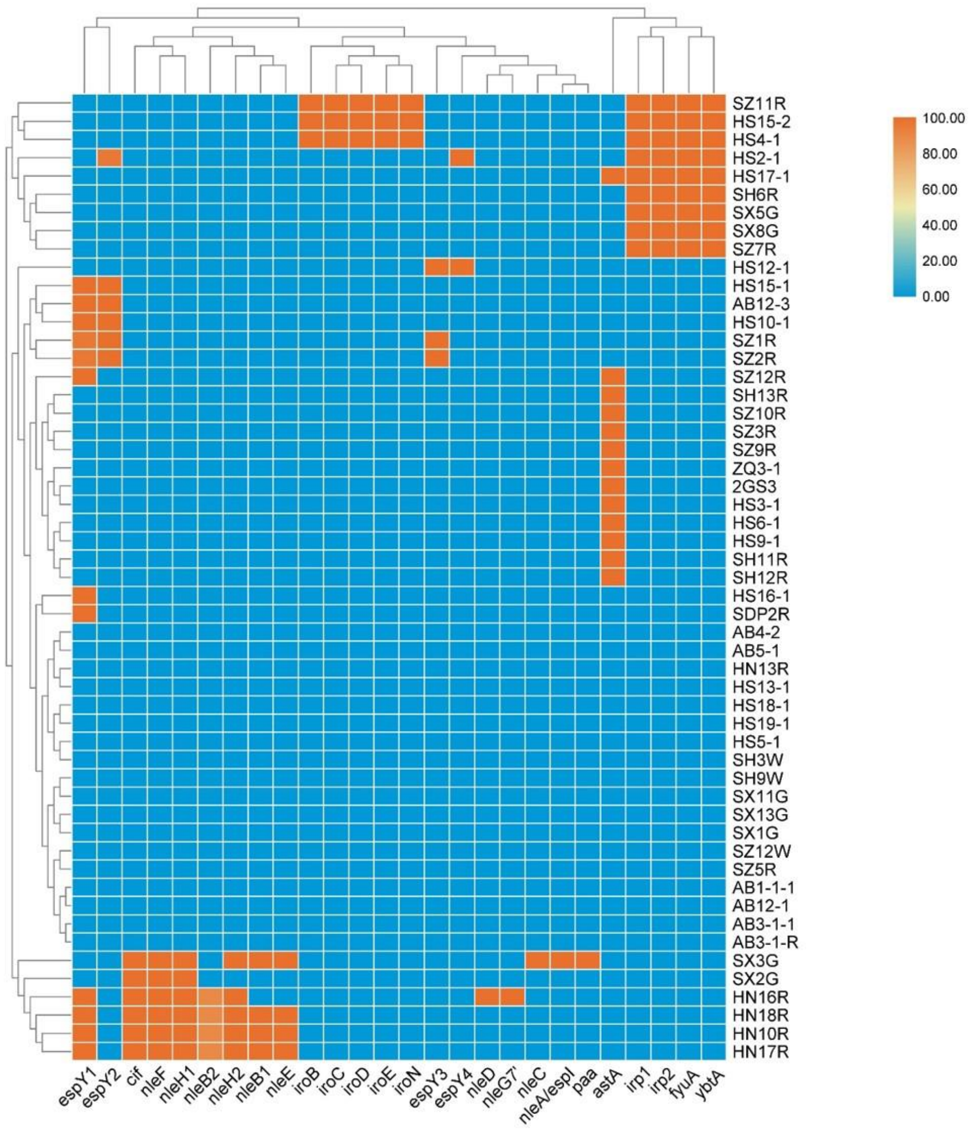

Fig. S1. The heat map of virulence genes of *tet*(X4)-positive *E. coli*. The darker the orange, the higher the similarity.

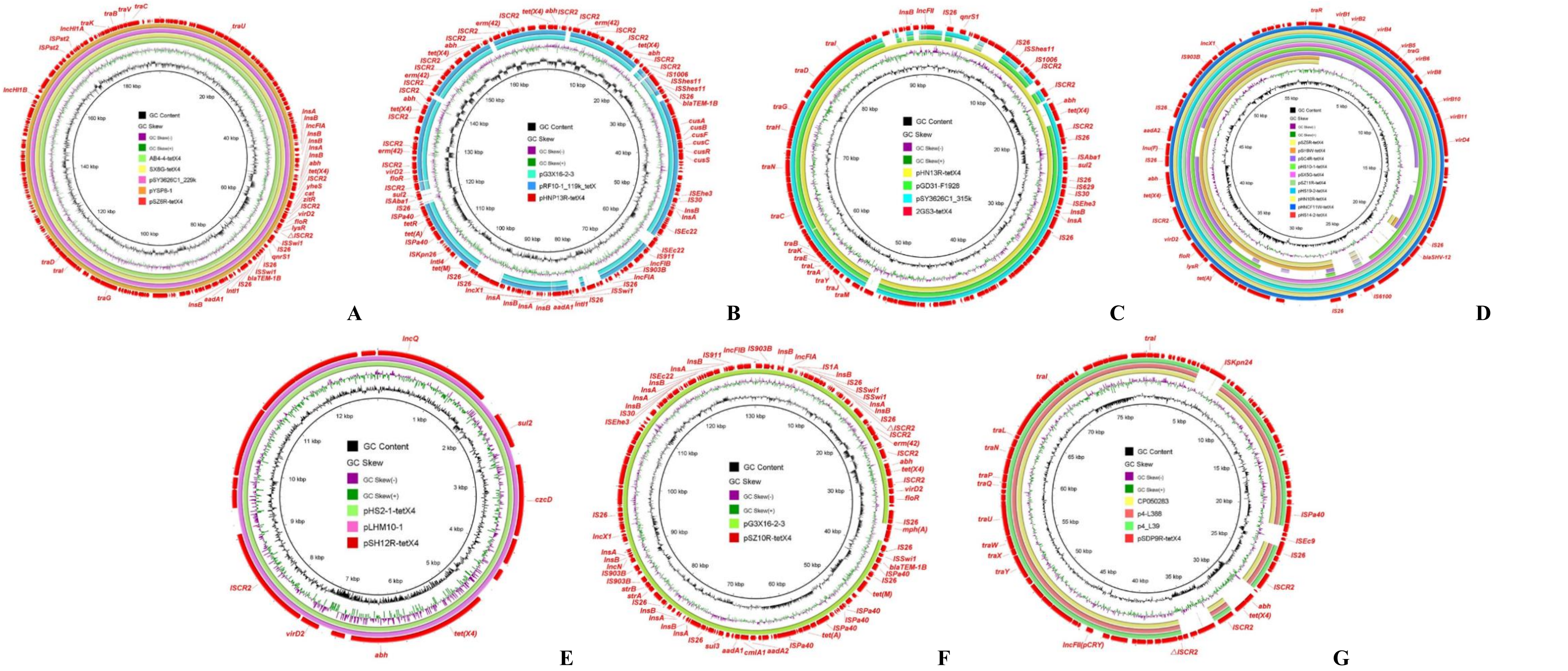

Fig. S2. Circular comparison of different *tet*(X4)-bearing plasmids with similar online plasmids. a, b, c, d, e and f represented different *tet*(X4)-bearing plasmids with various replicons IncFIA/IncHI1B/IncHIA, IncFIA/IncHI1B/IncX1, IncFII, IncQ, IncX1, IncFIA/IncHI1B/IncX1/IncN, IncFII.

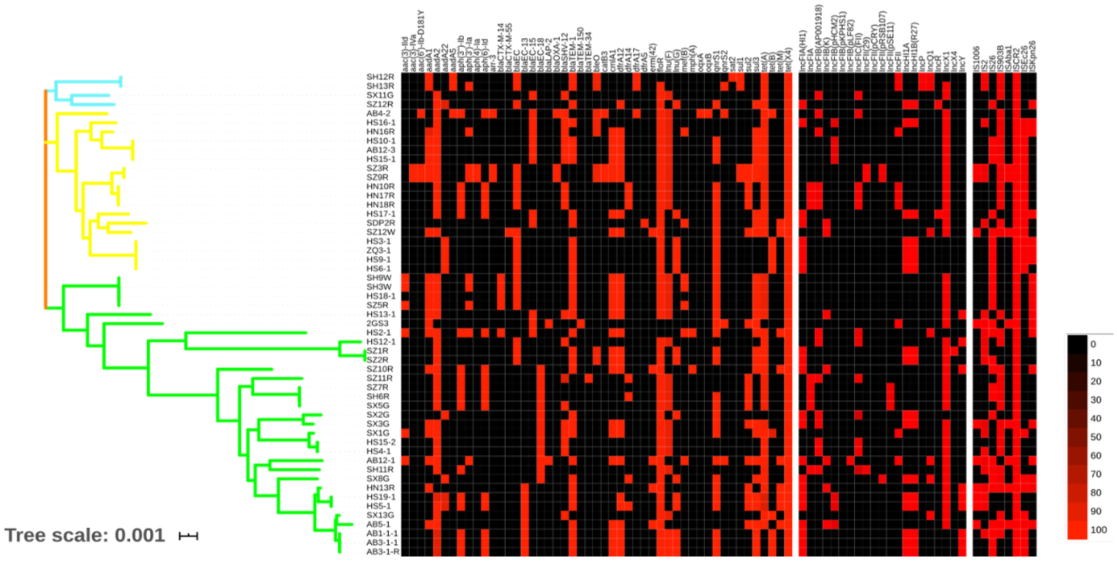

Fig. S3. Phylogenetic tree of 53 *tet*(X4)-positive *E. coli* from pork samples. The right part represents the heatmap of antimicrobial resistance genes, type of the plasmid replicon and inserted sequence in *tet*(X)-positive *E. coli* strains.

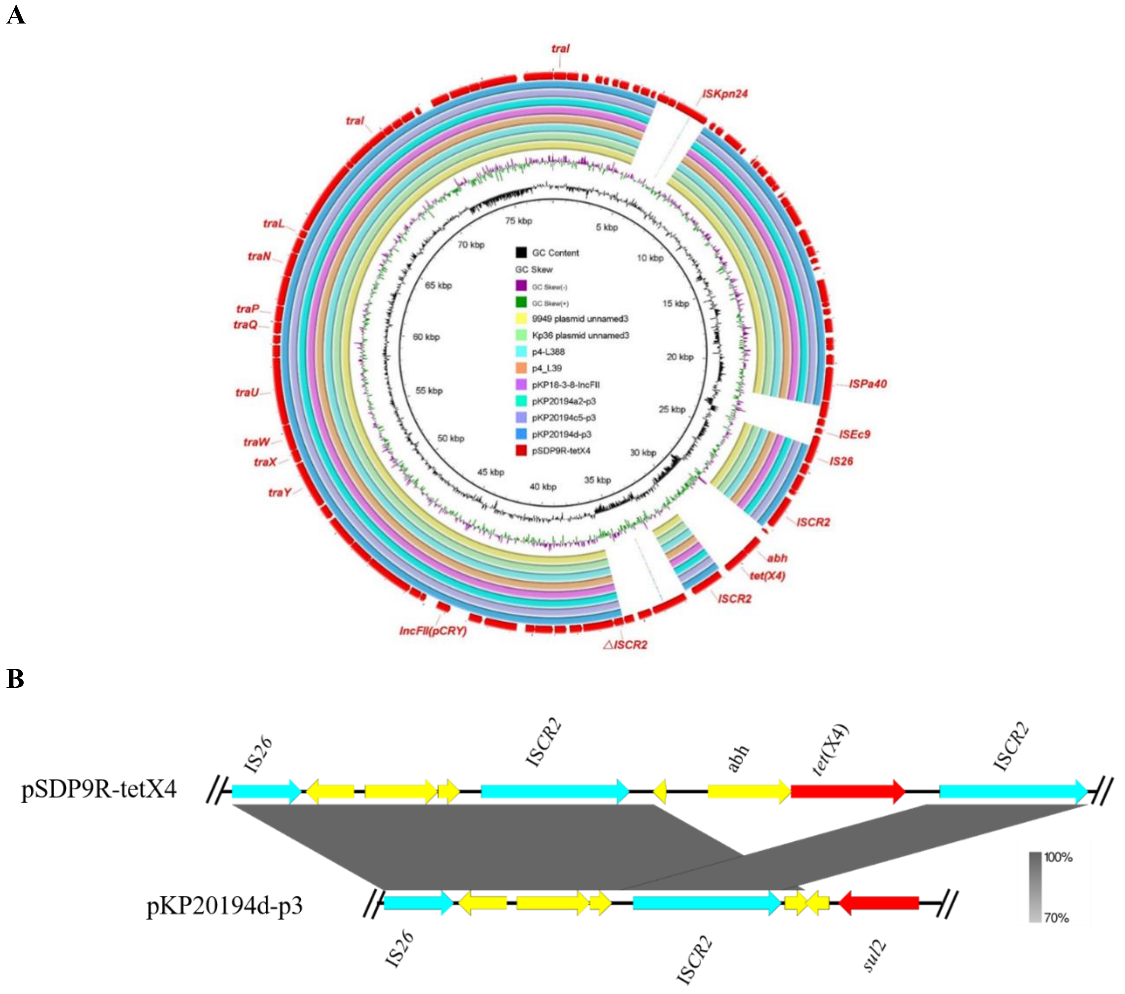

Fig. S4. Structures of the pSDP9R-tetX4 and other similar plasmids. (A) Circular comparison between the *tet*(X4)-bearing IncFII (pCRY) plasmid pSDP9R-tetX4 and other IncFII (pCRY) plasmids in the NCBI nr database. The other IncFII (pCRY) plasmids were all isolated from clinical strains. (B) Comparative analysis of the structures of the *tet*(X4)-bearing region found in plasmid pSDP9R-tetX4 and pKP20194d-p3 (NZ_CP054735).

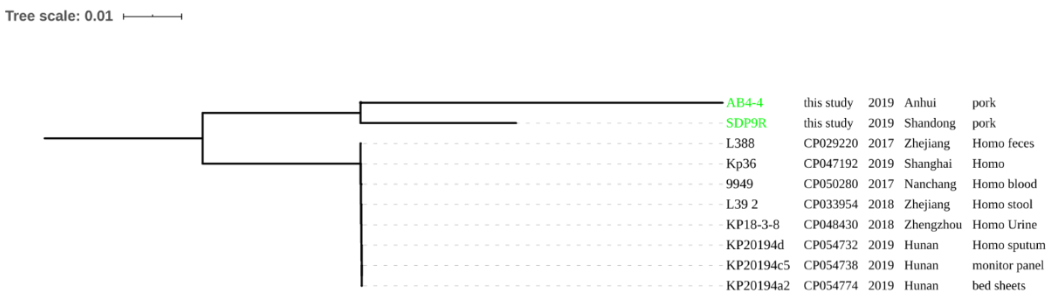

Fig. S5. Phylogenetic tree of two *tet*(X4)-positive *K. pneumonia* strains and other *tet*(X4)-negative *K. pneumonia* retrieved from NCBI genome database.

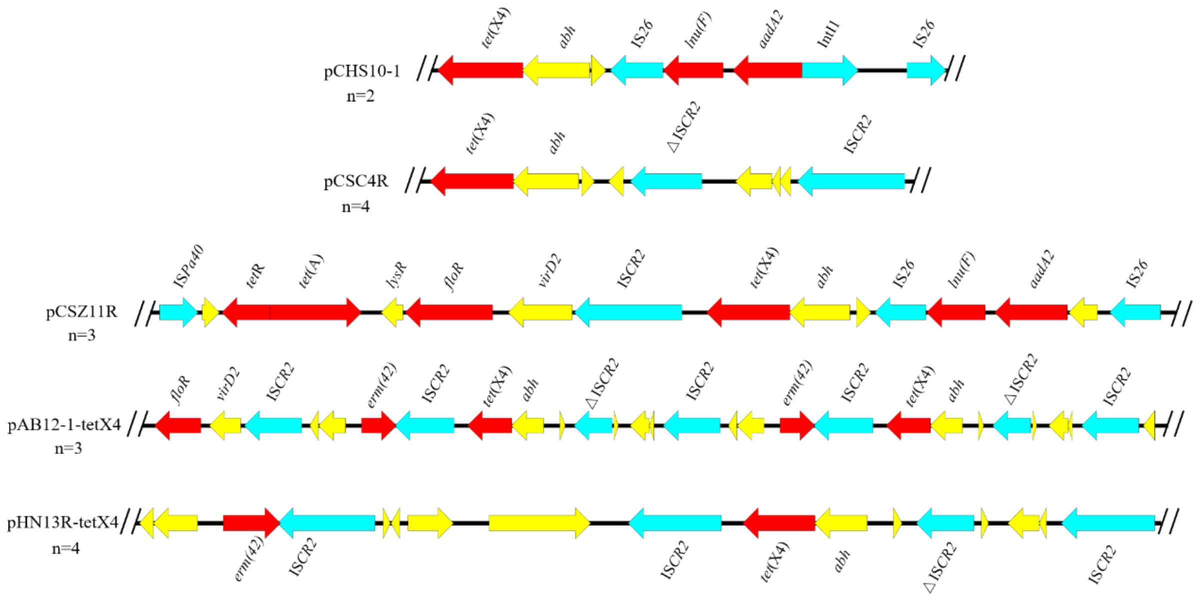

Fig. S6. Different copy number variations of *tet*(X4)-bearing repeat region. Copy number variations of *tet*(X4)-bearing tandem repeat structures based on Nanopore long-read analysis of single DNA molecules.

A

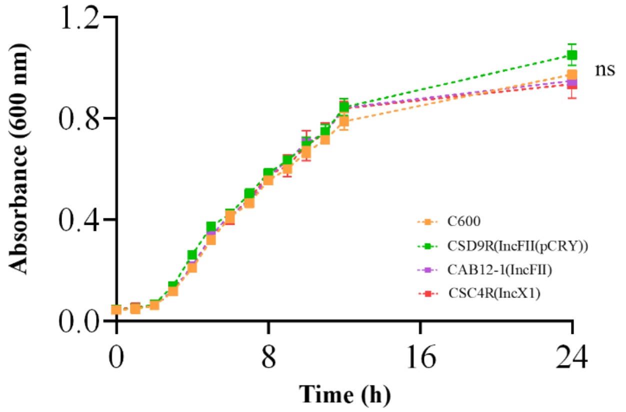

B

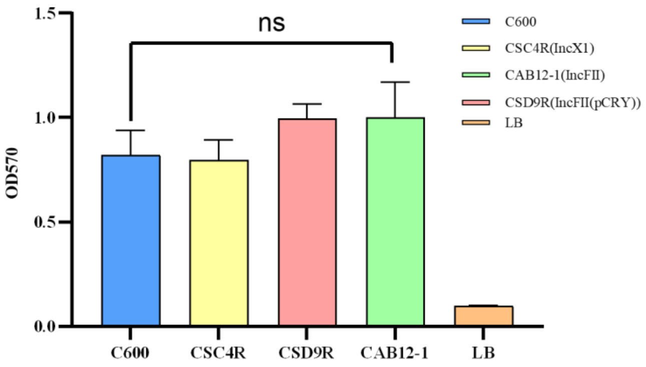

Fig. S7. Growth kinetics and Biofilm formation ability of different transconjugants of different plasmid replicon types. ns means no significant differences.

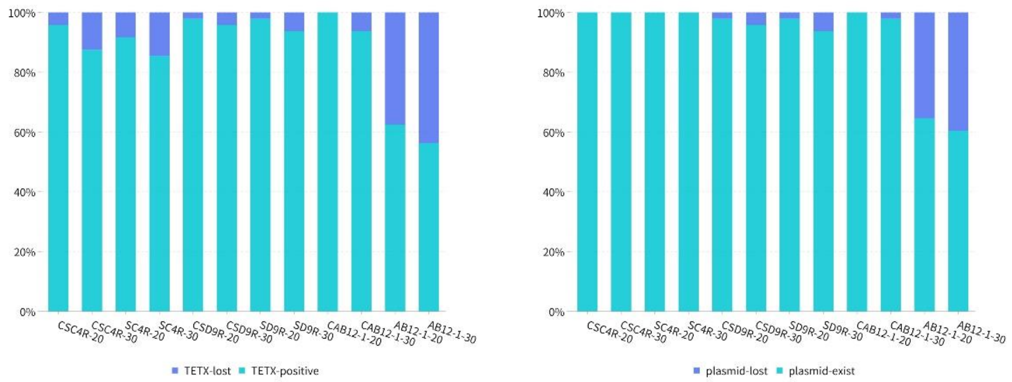

Fig. S8. The stability of *tet*(X4) and *tet*(X4)-positive plasmids of different plasmid replicon types in the strains and their transconjugants under tigecycline-free medium. Without tigecycline drug pressure, the thirtieth generation of each strain were all detected the phenomenon that *tet*(X4) lost.

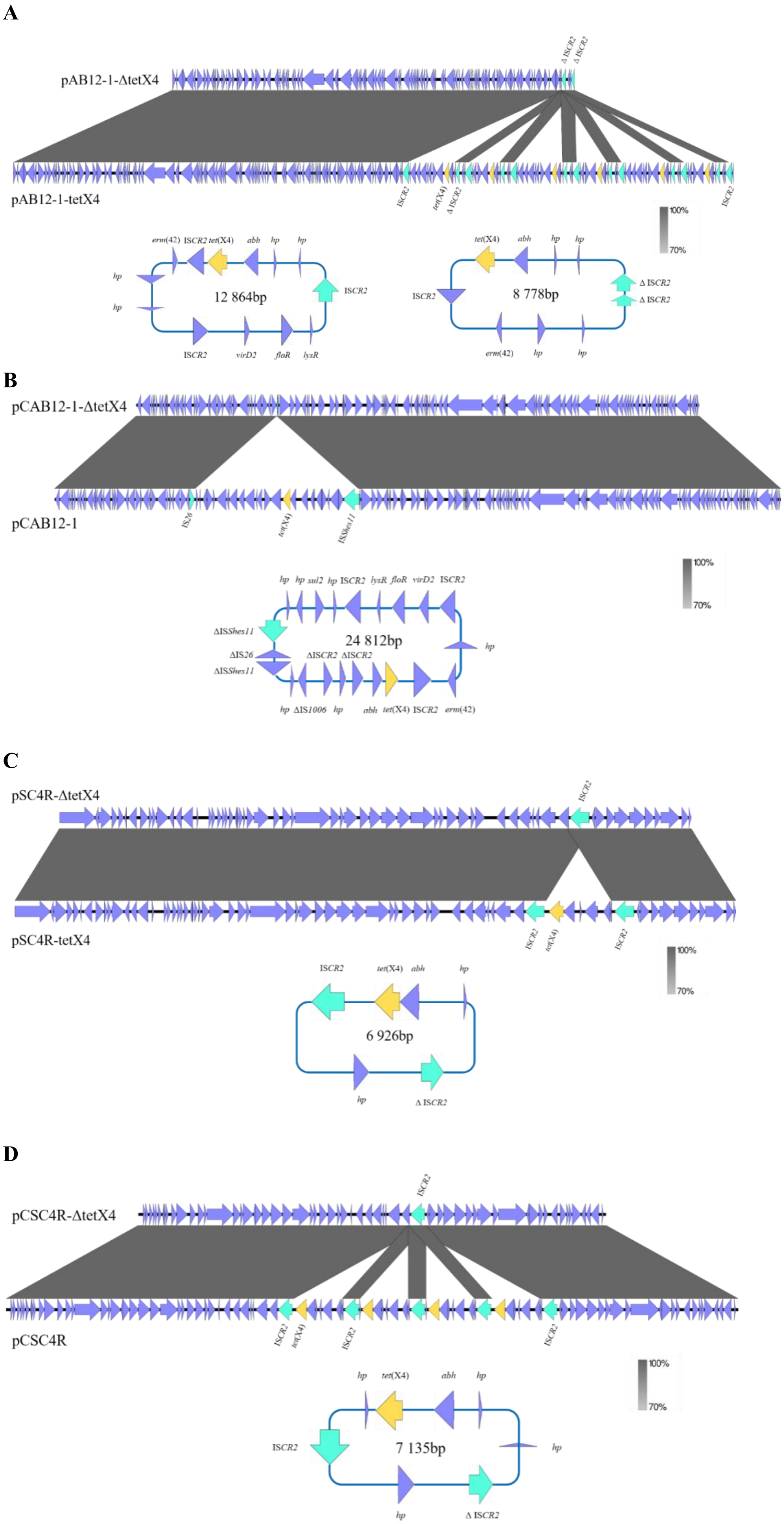

Fig. S9. Linear comparison of the genetic context of *tet*(X4)-positive plasmid with it *tet*(X4)-lost plasmid. The gray regions indicate the homologous region between plasmid regions.

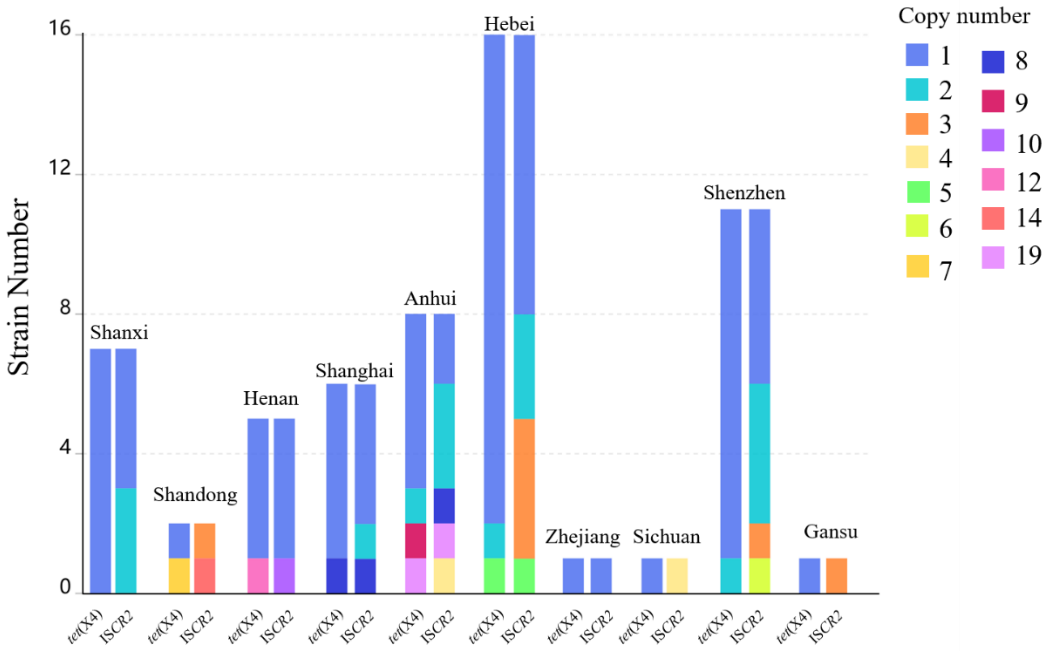

Fig. S10. The relationship between different copy numbers of IS*CR2* and *tet*(X4) in different regions. Different colors represent different copy numbers of *tet*(X4) gene and IS*CR2*.

A

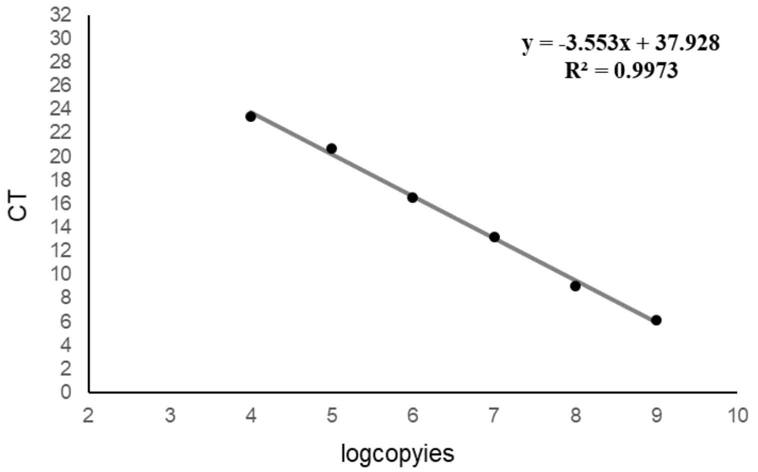

B

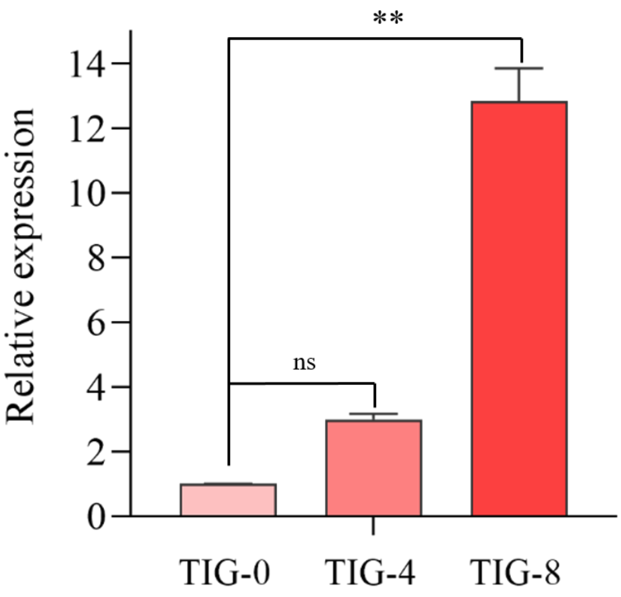

Fig. S11. The expression quantity of the *te*t(X4) gene. (A) Standard curves of the *tet*(X4) gene. Six-point standard curves with copy numbers ranging from 10^9 to 10^4 for qPCR were generated. (B) The relative expression of *tet*(X4) gene under different concentrations of tigecycline. *: P < 0.05; **: P < 0.01; ***: P < 0.001.
